# Recombinant shingles vaccination and the risk of cardiovascular events

**DOI:** 10.1038/s41591-026-04606-0

**Published:** 2026-08-26

**Authors:** Fabiana Corsi-Zuelli, Fang Li, Rachel Upthegrove, John A. Todd, Betty Raman, Paul J. Harrison, Maxime Taquet

**Affiliations:** 1 Department of Psychiatry, University of Oxford, Warneford Hospital, Oxford, UK; 2 Institute for Mental Health, University of Birmingham, Birmingham, UK; 3 Early Intervention Service, Birmingham and Solihull Mental Health Foundation Trust, Birmingham, UK; 4 Oxford Health NHS Foundation Trust, Oxford, UK; 5 Centre for Human Genetics, Nuffield Department of Medicine, Bukhman Centre for Research Excellence in Type 1 Diabetes, NIHR Biomedical Research Centres, University of Oxford, Oxford, UK; 6 Division of Cardiovascular Medicine, Radcliffe Department of Medicine, University of Oxford, Oxford, UK; 7 Oxford University Hospitals NHS Foundation Trust, Oxford, UK

**Keywords:** Preventive medicine, Cardiovascular diseases, Epidemiology, Viral infection

## Abstract

The recombinant herpes zoster (shingles) vaccine may reduce risks of cardiovascular disease. However, existing studies have compared vaccine recipients with non-recipients and, thus, are susceptible to confounding. To mitigate these biases, we conducted a natural experiment created by the rapid transition from the live attenuated to the recombinant shingles vaccine in the United States. We compared incidences of a composite cardiovascular endpoint (ischemic heart disease, heart failure or ischemic stroke) among adults aged ≥60 years vaccinated immediately before versus immediately after this transition. The recombinant vaccine was associated with a 9% decrease in cardiovascular burden over 7 years (restricted mean time lost (RMTL) ratio = 0.91, 95% confidence interval (CI): 0.88−0.95). The association attenuated over time and was significant for ischemic heart disease (10% decrease in burden; RMTL ratio = 0.90, 95% CI: 0.87−0.94) and heart failure (12% decrease in burden; RMTL ratio = 0.88, 95% CI: 0.83−0.93) in both sexes and ischemic stroke in males (12% decrease; RMTL ratio = 0.88, 95% CI: 0.78−0.98). An association was also seen for atrial fibrillation (7% decrease in burden; RMTL = 0.93, 95% CI: 0.88−0.98) but not other cardiac, peripheral and cerebrovascular outcomes. Results were consistent in secondary analyses. These results justify clinical trials and mechanistic studies to investigate potential cardioprotective effects of shingles vaccines.

## Abstract (web summary)

A natural experiment comparing recipients of the live attenuated versus recombinant herpes zoster vaccines found that the recombinant vaccine was associated with a lower incidence of cardiovascular events across 7 years of follow-up.

## Main

Herpes zoster (shingles) is a painful condition caused by reactivation of latent varicella-zoster virus (that causes chickenpox)^1^. Shingles vaccination is recommended in many countries to mitigate the deleterious consequences of the infection in older adults.

There has been recent interest in the possibility that the recombinant shingles vaccine may reduce the risk of cardiovascular disease^2–4^. However, existing studies have relied on comparisons between vaccinated and unvaccinated individuals. This design is prone to ‘healthy-vaccinee’ bias because people who choose to get vaccinated differ systematically from those who choose not to in ways that are not measured in the data and cannot be accounted for in the analysis.

**Fig. 1:**
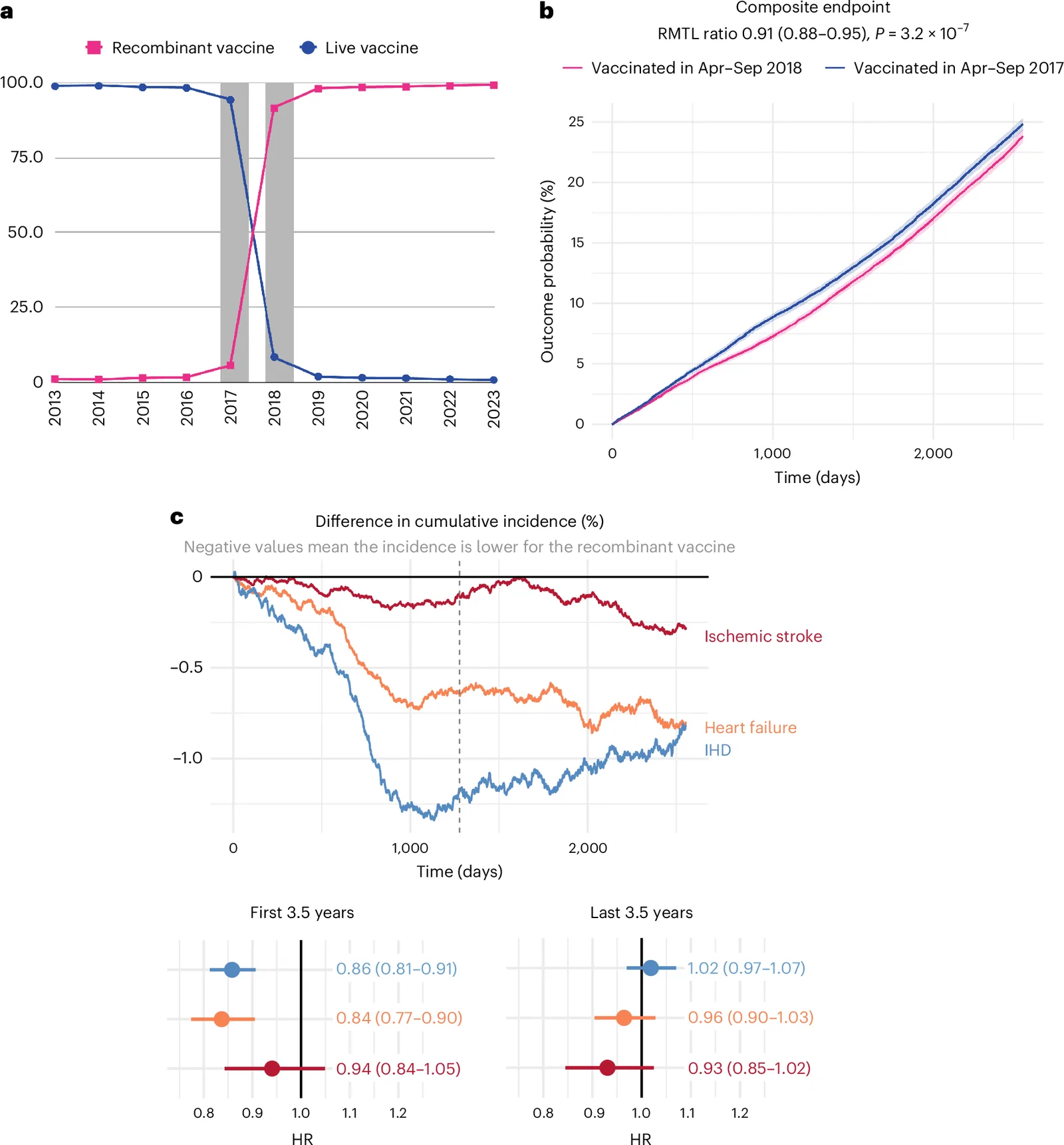
Association between recombinant shingles vaccine and risk of cardiovascular outcomes within 7 years of vaccination. **a**, Proportion (in %) of vaccine type received, showing the step change that occurred in October 2017. The exposure windows used in the primary analysis are shown in gray. **b**, Kaplan−Meier curves of the cumulative incidence for the cardiovascular composite endpoint within 7 years of vaccination between those vaccinated in April−September 2017 (predominantly live vaccine) and those vaccinated in April−September 2018 (predominantly recombinant vaccine) (*n* = 36,460 in each cohort). Shaded areas represent 95% CIs of the cumulative incidence. Solid lines show the Kaplan−Meier estimate of the cumulative incidence (the measure of center). Between-cohort differences were assessed using the RMTL ratio with a two-sided *z*-test (survRM2 R package), without adjustment for multiple comparisons. **c**, Temporal evolution of risk for the individual components of the composite cardiovascular endpoint after shingles vaccination in 2018 compared to 2017—differences in cumulative incidence over time (top panel) and piecewise constant hazard ratios (HRs) for the first and last 3.5 years of follow-up (bottom panels) (*n* = 36,460 in each cohort). In the top panel, the line shows the difference in Kaplan−Meier cumulative incidence between cohorts (the measure of center) and the shaded band its 95% CI; in the bottom panels, points show the piecewise constant HR (the measure of center) and error bars its 95% CI. *P* values are two-sided. Apr, April; IHD, ischemic heart disease; Sep, September.

Here we used a natural experiment arising from the rapid uptake of the recombinant vaccine and the concurrent discontinuation of the live attenuated vaccine after October 2017 (Fig. 1a). Because there is no obvious reason for systematic differences between people who were vaccinated on either side of this time cutoff, this design mitigates biases that affect conventional cohort studies. By comparing cohorts of individuals who have both chosen to be vaccinated against shingles (with the live vaccine serving as a control given its lack of association with cardiovascular outcomes^5^), this study design also eliminates the healthy-vaccinee bias. Using a similar design, we previously provided evidence that recombinant shingles vaccination may lower the risk of dementia^6^.

We compared cardiovascular outcomes among adults ≥60 years of age vaccinated against shingles within the 6 months preceding the transition from live to recombinant shingles vaccination (1 April to 30 September 2017) and the same period (to eliminate seasonal effects^7^) a year later (1 April to 30 September 2018) when the recombinant shingles vaccine was predominantly used (Fig. 1a). Our primary outcome was a composite cardiovascular endpoint comprising ischemic heart disease, ischemic stroke and heart failure. We used propensity score matching to further control for drifts in the characteristics of the vaccinated population.

A total of 36,460 individuals who received their shingles vaccine between April and September 2018 (93.5% received the recombinant vaccine) were propensity score matched to 36,460 individuals who received their shingles vaccine between April and September 2017 (98.6% received the live vaccine) (Supplementary Table 1).

**Extended Data Fig. 1:**
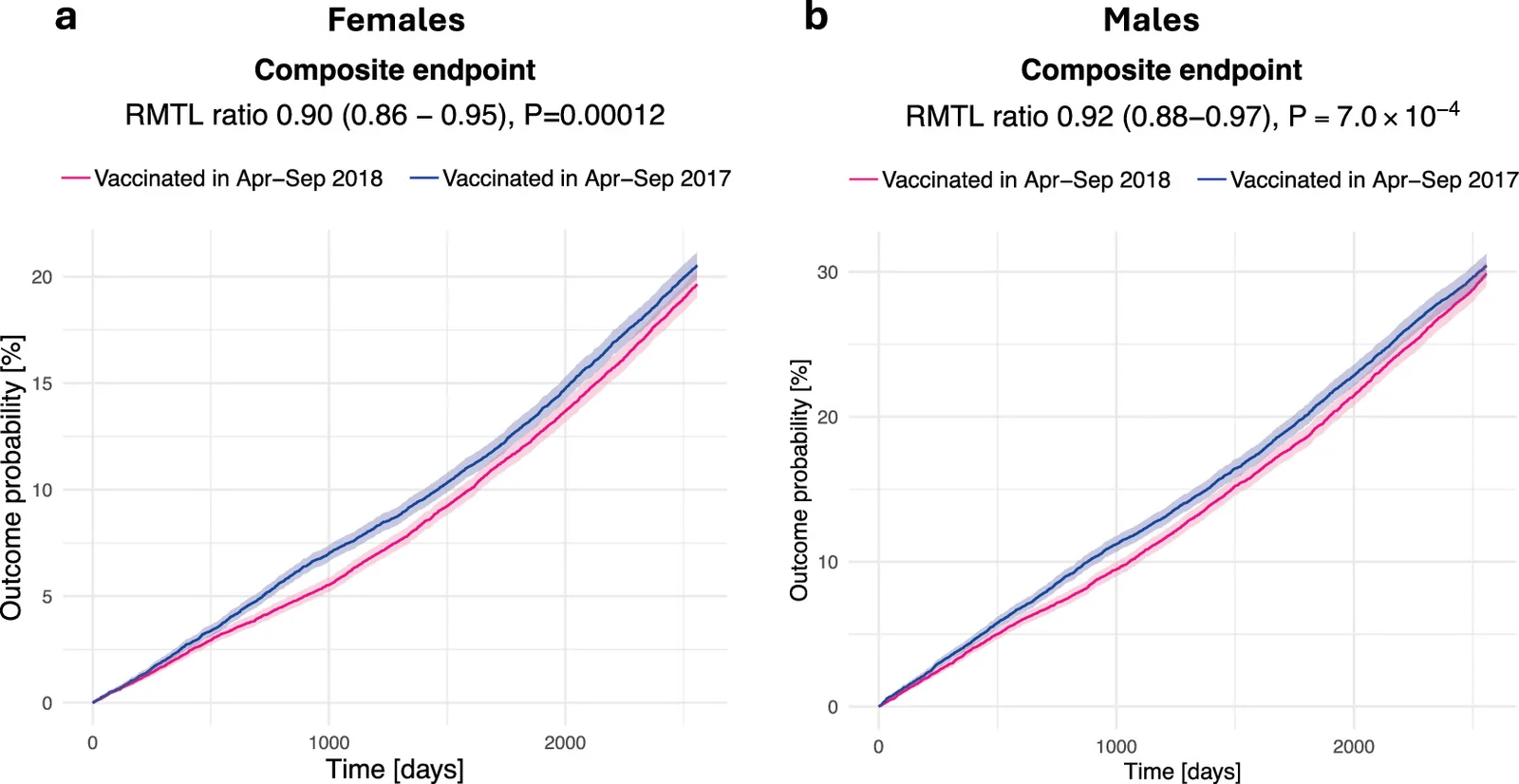
Association between the recombinant shingles vaccine and risk of cardiovascular events within 7 years of vaccination, stratified by sex. Curves represent the Kaplan–Meier estimates of the cumulative incidence for the cardiovascular composite endpoint within 7 years of vaccination among (**a**) females (n = 20,056 in each cohort) and (**b**) males (n = 16,300 in each cohort). Shaded areas around the curves represent 95% confidence intervals, and the solid Kaplan–Meier curve is the measure of centre. Two-sided P values were obtained using the z-test defined in the survRM2 package in R.

Individuals in the group who predominantly received the recombinant vaccine were at a lower risk of cardiovascular events over the next 7 years than those in the group who predominantly received the live vaccine: RMTL ratio = 0.91, 95% CI: 0.88−0.95, *P* < 0.001, translating into 9% more time lived diagnosis free (Fig. 1b and Table 1). The *E* value was 1.42, indicating that an unmeasured confounder would need to be 1.42 times more common in those vaccinated in 2017 than in those vaccinated in 2018 and to be associated with a 1.42-fold increased risk of cardiovascular outcomes after matching for all other covariates to have a chance to explain away our finding. The association was found to hold in both females and males (Table 1 and Extended Data Fig. 1) with no moderation by sex (permutation test: *P* = 0.53; Supplementary Table 2).

**Table 1:** Summary of results

	*n*	RMTL ratio (95% CI)	*P* value
Primary outcomes			
Cardiovascular composite endpoint	36,460	0.91 (0.88−0.95)	3.2 × 10^−7^
Females	20,056	0.90 (0.86−0.95)	0.00012
Males	16,300	0.92 (0.88−0.97)	3.2 × 10^−4^
Ischemic heart disease	36,460	0.90 (0.87−0.94)	6.0 × 10^−7^
Females	20,056	0.91 (0.85−0.96)	0.00012
Males	16,300	0.92 (0.88−0.97)	0.002
Heart failure	36,460	0.88 (0.83−0.93)	7.4 × 10^−6^
Females	20,056	0.84 (0.77−0.91)	1.4 × 10^−5^
Males	16,300	0.91 (0.84−0.98)	0.01
Ischemic stroke	36,460	0.95 (0.87−1.03)	0.18
Females	20,056	0.92 (0.82−1.03)	0.16
Males	16,300	0.88 (0.78−0.98)	0.02
Secondary outcomes			
Atrial fibrillation	36,460	0.93 (0.88−0.98)	0.0072
STEMI	36,460	0.88 (0.75−1.04)	0.13
Myocarditis	36,460	0.97 (0.56−1.69)	0.92
Peripheral arterial disease	36,460	1.07 (0.78−1.47)	0.67
Transient ischemic attack	36,460	0.95 (0.86−1.06)	0.35
Hemorrhagic stroke	36,460	1.08 (0.93−1.25)	0.33
Sensitivity analyses			
Composite negative control outcome	36,460	1.07 (0.97−1.17)	0.16
Composite endpoint or death	36,460	0.92 (0.89−0.95)	9.5 × 10^−7^
Shingles (positive control)	36,460	0.73 (0.65−0.82)	5.2 × 10^−8^
Aligned follow-up	36,460	0.92 (0.89−0.96)	1.9 × 10^−5^
Follow-up restricted to before the COVID-19 pandemic	36,460	0.89 (0.82−0.97)	0.0068
Testing secular trend in cardiovascular endpoint	360,753	1.01 (0.99−1.03)	0.48
Alternative covariates ascertainment window			
1 year	35,340	0.94 (0.92−0.97)	7.5 × 10^−6^
2 years	35,193	0.94 (0.91−0.96)	3.9 × 10^−6^
5 years	35,314	0.94 (0.92−0.97)	9.1 × 10^−6^
10 years	35,226	0.94 (0.92−0.97)	6.1 × 10^−6^
Competing risk analysis, AJ-RMTL ratios			
Cardiovascular composite endpoint	36,460	0.90 (0.87−0.93)	8.4 × 10^−9^
Non-cardiovascular death	36,460	1.00 (0.91−1.14)	0.86

*n*, number of individuals in each cohort after matching. The *P* values correspond to the *z*-test defined in the survRM2 package in R. All *P* values are two-sided. Note that the sample size for males and females does not add up to the total sample size because the latter includes people with unknown sex and because, for sex-stratified analyses, matching is achieved in each sex category independently.

**Extended Data Fig. 2:**
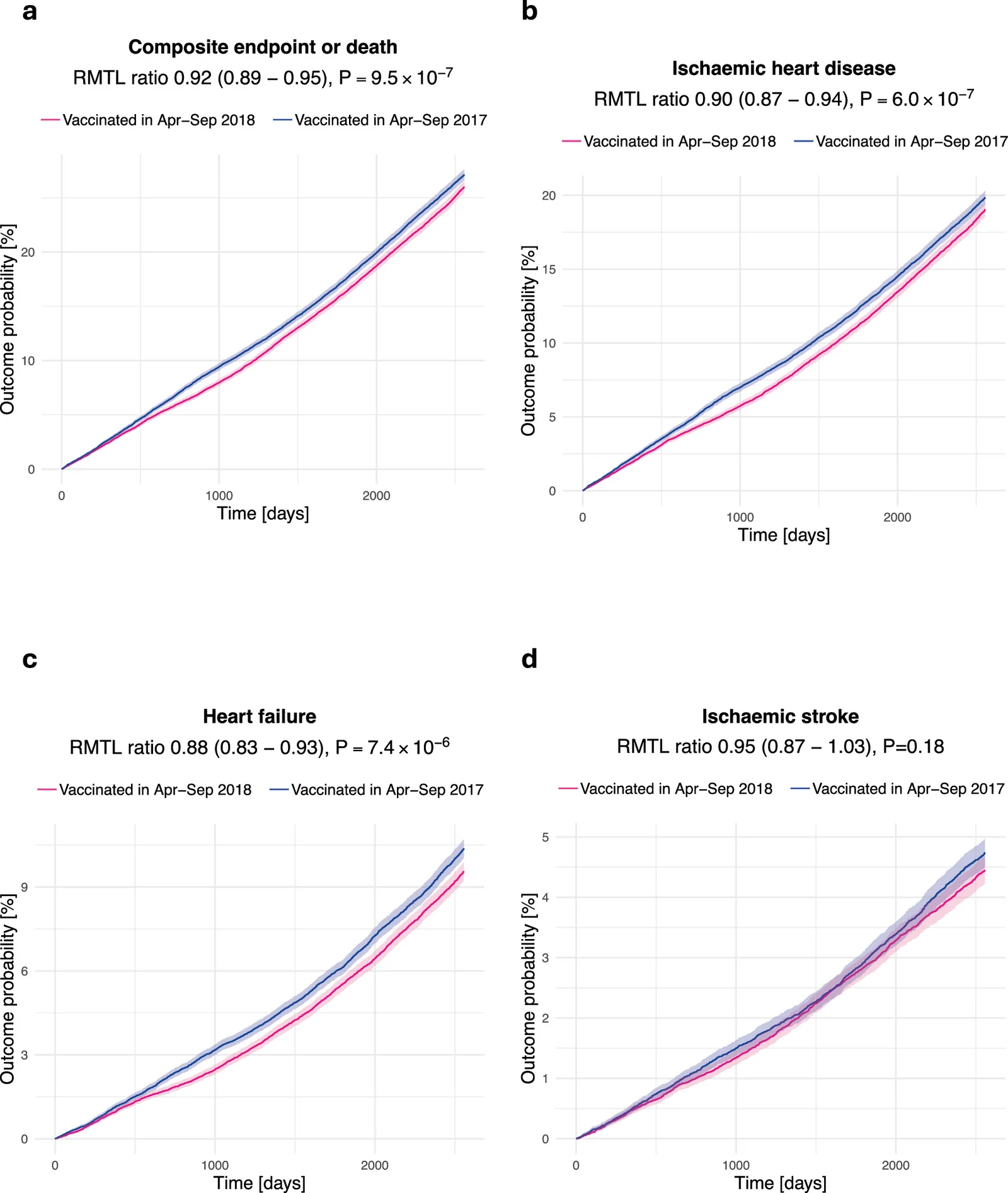
Association between the recombinant shingles vaccine and risks of the individual components of the cardiovascular endpoint within 7 years of vaccination. Curves represent the Kaplan–Meier estimates of the cumulative incidence of (**a**) the cardiovascular composite endpoint or death, (**b**) ischaemic heart disease, (**c**) heart failure and (**d**) ischaemic stroke. Shaded areas around the curves represent 95% confidence intervals; the solid Kaplan–Meier curve is the measure of centre. n = 36,460 in each cohort. Two-sided P values were obtained using the z-test defined in the survRM2 package in R.

**Extended Data Fig. 3:**
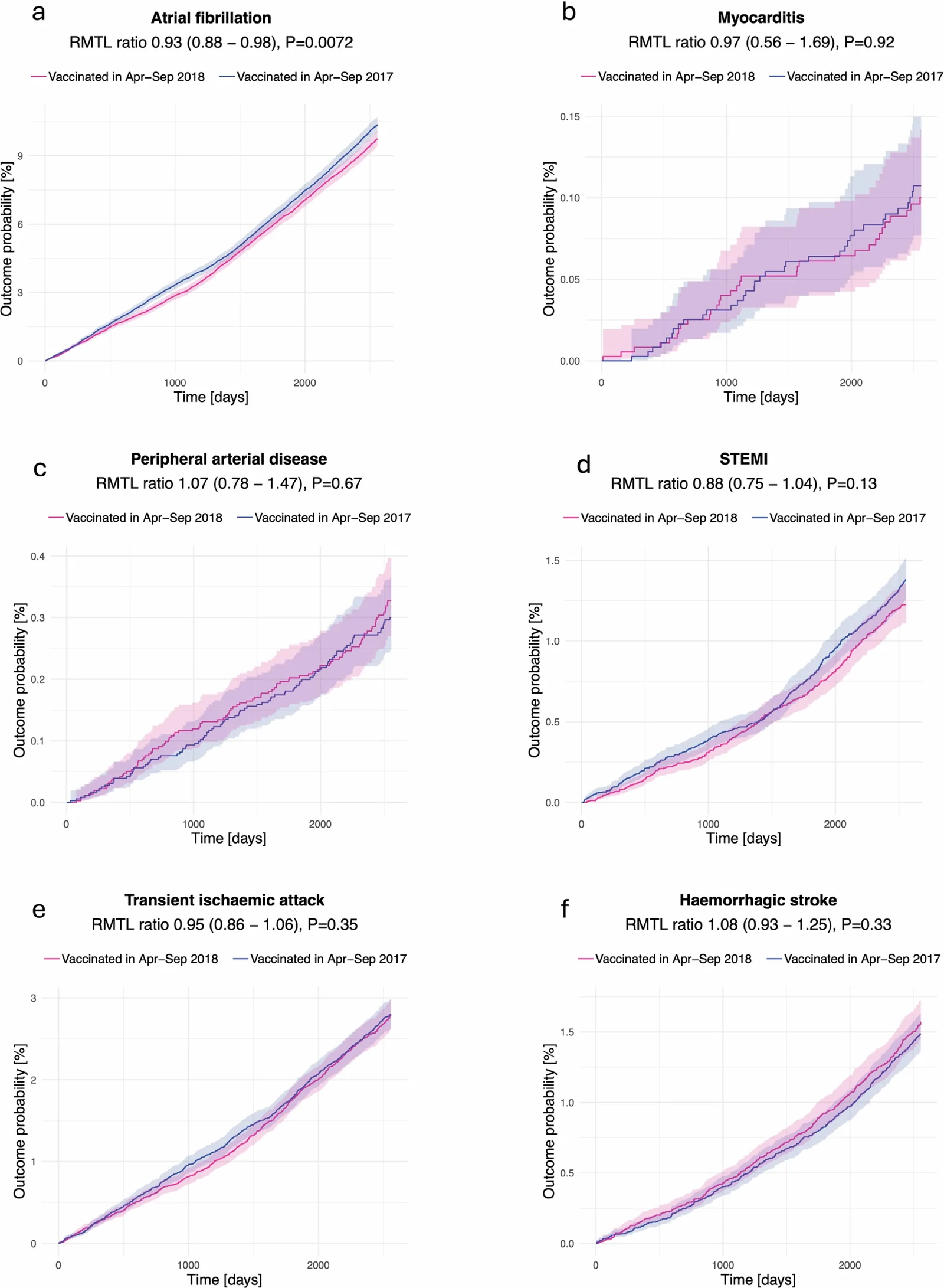
Association between the recombinant shingles vaccine and risks of secondary outcomes within 7 years of vaccination. Curves represent the Kaplan–Meier estimates of the cumulative incidence of (**a**) atrial fibrillation, (**b**) myocarditis, (**c**) peripheral arterial disease, (**d**) ST-elevation myocardial infarction (STEMI), (**e**) transient ischaemic attack and (**f**) haemorrhagic stroke; n = 36,460 in each cohort. Shaded areas around the curves represent 95% confidence intervals, and the solid Kaplan–Meier curve is the measure of centre. Two-sided P values were obtained using the z-test defined in the survRM2 package in R.

Those who predominantly received the recombinant shingles vaccine had lower incidences of the composite of cardiovascular events or death, ischemic heart disease and heart failure, with males also having a lower risk of ischemic stroke (Table 1 and Extended Data Fig. 2). Those who predominantly received the recombinant vaccine were also at a lower risk of atrial fibrillation but not myocarditis, peripheral arterial disease, hemorrhagic stroke or transient ischemic attack; association with ST-elevation myocardial infarction (STEMI) was of similar magnitude as for ischemic heart disease and heart failure but did not reach statistical significance (Table 1 and Extended Data Fig. 3).

**Extended Data Fig. 4:**
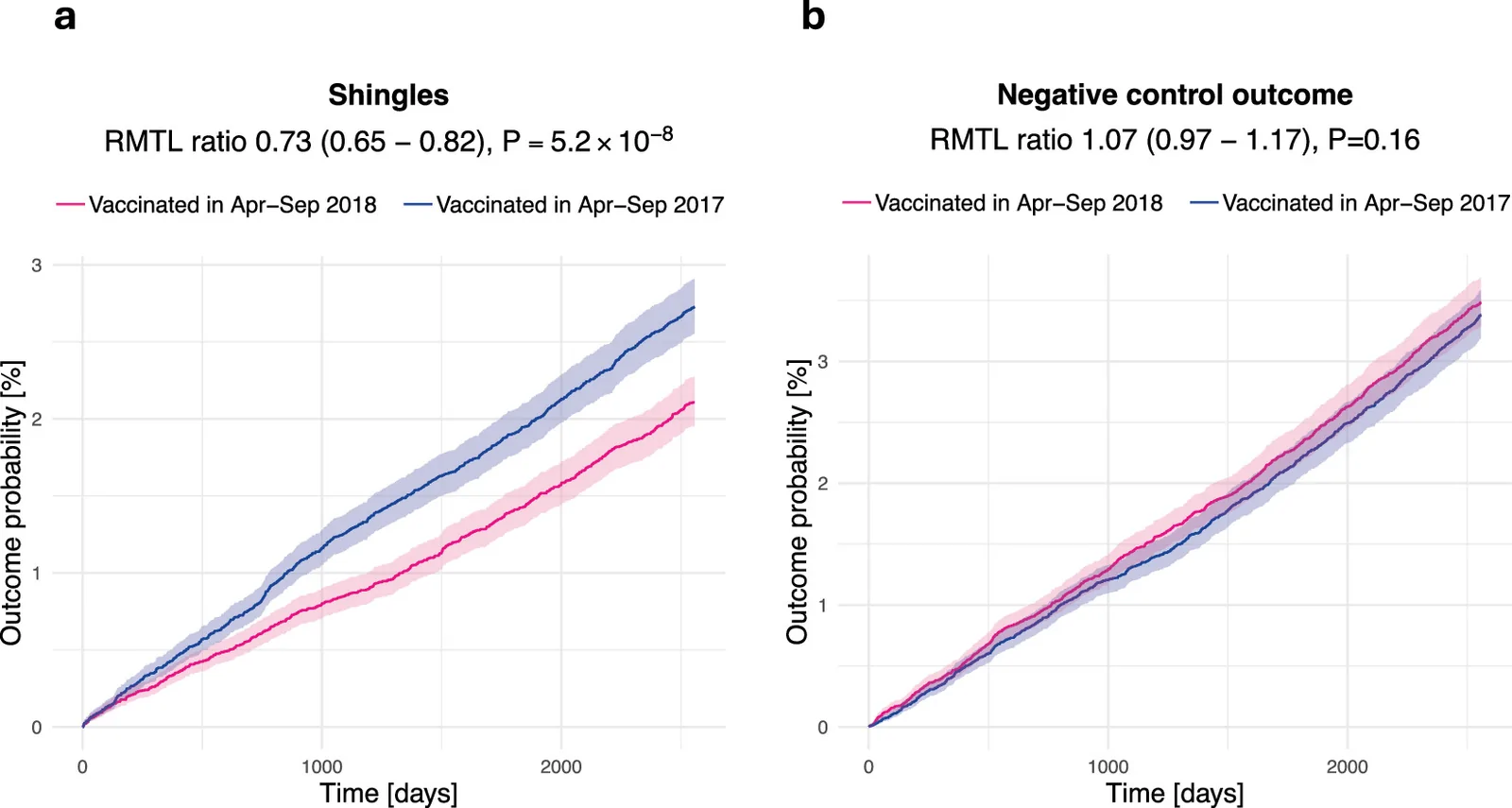
Association between the recombinant shingles vaccine and risks of the positive and negative control outcomes within 7 years of vaccination. Curves for (**a**) shingles (positive control) and (**b**) the negative control outcome. Shaded areas around the curves represent 95% confidence intervals; the solid Kaplan–Meier curve is the measure of centre. n = 36,460 in each cohort. Two-sided P values were obtained using the z-test defined in the survRM2 package in R.

The association remained similar when accounting for death as a competing risk (and there was no association with non-cardiovascular death; Table 1 and Supplementary Table 3); when the follow-up period was entirely before the COVID-19 pandemic (Supplementary Table 4); when the amount of follow-up time was matched between cohorts (Supplementary Table 5); and when different time windows were used to ascertain covariates (Table 1). In the year before vaccination, the two cohorts did not differ in preventive healthcare use or in the risk of cardiovascular events (Supplementary Table 6). Likewise, there was no significant difference between cohorts during follow-up in terms of preventive health indicators (Supplementary Table 7). As expected, individuals who predominantly received the recombinant vaccine were at a lower risk of shingles and a similar risk of negative control outcome than those who predominantly received the live vaccine (Table 1 and Extended Data Fig. 4). There was no sign that secular trends in diagnostic rates confounded the results: those who received the tetanus, diphtheria and pertussis (TDaP) vaccine in April−September 2018 had a very similar risk of recorded cardiovascular events as those who received it during the same months in 2017 (Table 1).

**Extended Data Fig. 5:**
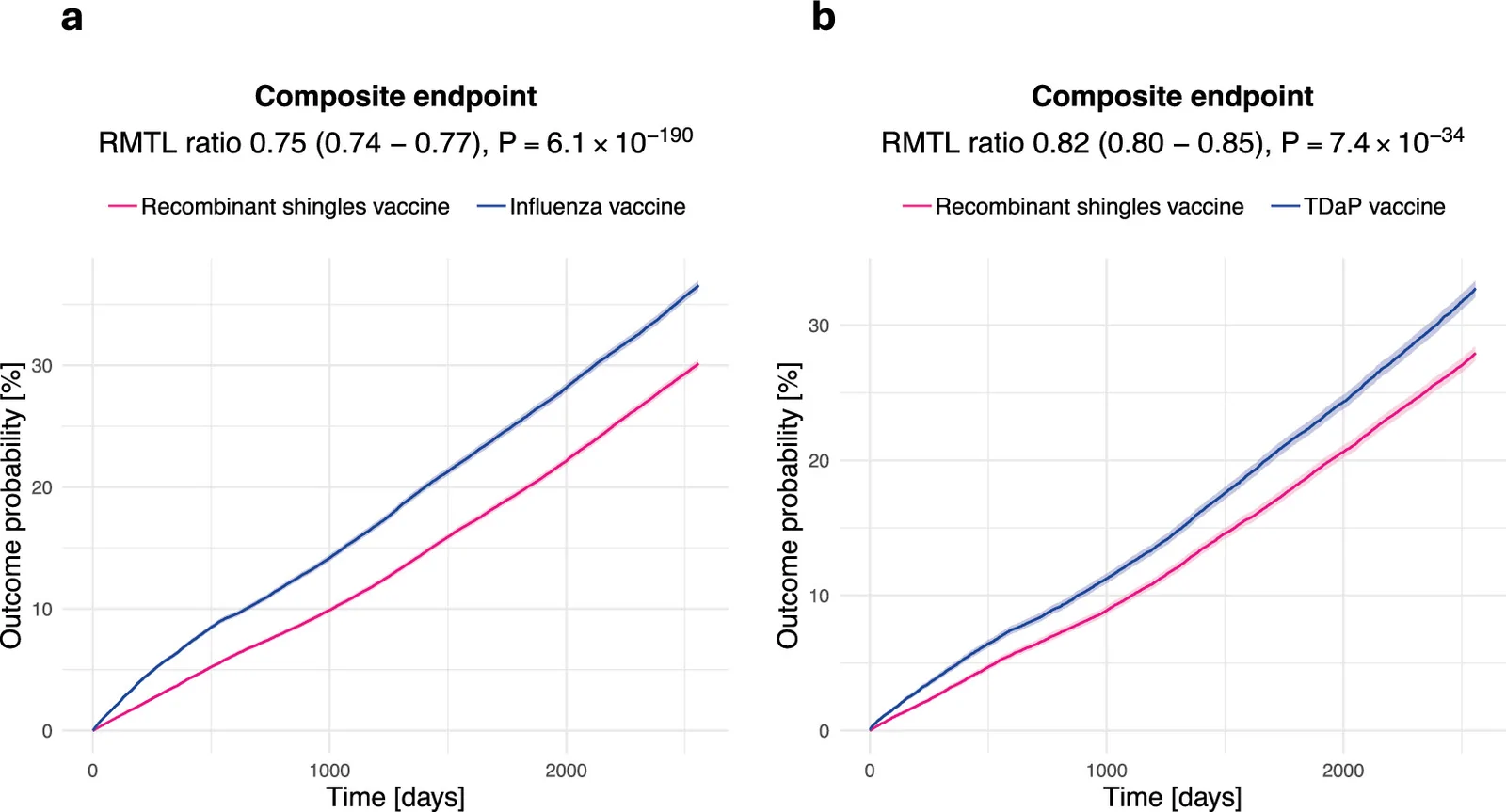
Kaplan–Meier curves comparing the recombinant shingles vaccine with the influenza and Tdap vaccines. Comparisons with (**a**) influenza (n = 94,923 in each cohort) and (**b**) Tdap (n = 39,087 in each cohort). The RMTL ratio and the two-sided P value (z-test defined in the survRM2 package in R) are reported above each panel. Shaded areas around the curves represent 95% confidence intervals.

The protective association against ischemic heart disease and heart failure was found to be present in the first half of follow-up (as seen in Fig. 1c by hazard ratios below 1 and an increasing difference in cumulative incidences) before attenuating in the second half (as seen in Fig. 1c by hazard ratios that are no longer significant and plateauing differences in cumulative incidence). The recombinant shingles vaccine was also associated with a lower risk of the primary endpoint and each of its components, when compared to the influenza and TDaP vaccines (Extended Data Fig. 5 and Supplementary Table 8), although this was based on conventional matched cohort comparisons that are more prone to bias. This might explain their greater effect sizes than those seen in the primary analysis.

The observed reduction of risk is clinically meaningful: if confirmed in clinical trials, a 0.8% absolute difference in cumulative incidences of ischemic heart disease and heart failure among adults over 60 years of age would translate into hundreds of thousands of cases prevented in the United States alone.

The stronger association observed for ischemic heart disease and heart failure than for ischemic stroke may indicate a cardiac-specific pattern. This would be notable given that both strokes and ischemic heart disease often result from ischemic injury to the vascular endothelium. One possible explanation is that structural and molecular differences between the cerebral and coronary arteries^8^, including their responses to immune perturbation^9^, modulate the effect of the recombinant shingles vaccine on vascular health. Alternatively, the null finding for strokes may reflect limited statistical power, as ischemic strokes occur 2−4 times less frequently than ischemic heart disease or heart failure in this age group (Extended Data Fig. 2). Consistent with this interpretation, an association was observed among males, in whom stroke event rates were 15% higher.

Myocarditis can follow viral infections and vaccination and can be misdiagnosed as ischemic heart disease^10^. However, the lack of association with myocarditis and the association of similar magnitude with STEMI (although not reaching statistical significance) suggests that ischemic heart disease does not represent misdiagnosed myocarditis. There is a bidirectional association between atrial fibrillation and ischemic heart disease^11^. The protective association of the recombinant vaccine with atrial fibrillation is, therefore, not surprising and serves as an additional validation of our findings. Further studies should try and elicit whether the protective association against ischemic heart disease is mediated by protection against atrial fibrillation (which could explain the lack of association with peripheral arterial disease) or vice versa.

The biological mechanisms underlying these observations remain to be clarified experimentally. Although prevention of shingles might partly explain the association (given the association between shingles and cardiovascular risk^12^ and the greater efficacy of the recombinant vaccine), this explanation appears unlikely for three reasons. First, the absolute difference in shingles cases between vaccines was less than 0.5% at mid-follow-up (Extended Data Fig. 4a), compared to a 1.5% reduction in ischemic heart disease (Fig. 1c). Second, protection would be expected to emerge gradually as shingles cases accumulate in the comparator group rather than showing the early divergence observed here. Third, a natural experiment of live attenuated shingles vaccination compared to no vaccination did not show reduced risks of heart disease or stroke^5^. A more plausible explanation is that the recombinant vaccine may induce immune and endothelial changes that could be cardioprotective. For instance, the AS01 adjuvant it contains triggers sustained epigenetic and functional reprogramming of monocytes, including reduced interleukin 6 (IL-6) responses to Toll-like receptor activation^13^, and IL-6 inhibition is causally associated with lower cardiovascular risk^14^. Such immune changes might be time limited, consistent with the attenuation of the association in the second half of the follow-up and supporting studies investigating the effects of repeated vaccination at regular intervals.

This study has several limitations beyond those inherent to electronic health record (EHR) research (for example, unvalidated diagnoses and limited socioeconomic and lifestyle data). First, we did not assess the impact of multiple vaccine doses, as this was incompatible with a natural experiment design. For the same reason, we did not compare the recombinant vaccine with no vaccine, instead assuming that the live vaccine had no cardiovascular effect (based on another natural experiment^5^). If the live vaccine provides some cardiovascular protection, then our results would be underestimates of the true protective association of the recombinant vaccine. Second, outcomes were defined using recorded diagnoses, so undiagnosed cases were not captured. However, if similarly distributed between cohorts, this would not bias primary comparisons. Third, although our study is a natural experiment (in that treatment allocation is probabilistic and dependent on an external factor^15^) and thus less prone to bias than conventional cohort studies, it does not imply that associations are causal. Causality requires additional assumptions (outlined in the Methods alongside supporting evidence and discussion of remaining uncertainties). In particular, although population drifts in measured characteristics (for example, age) were adjusted for, changes in unmeasured characteristics cannot be excluded. Such unmeasured confounders are plausible given that the transition from the live to the recombinant vaccine followed updated recommendations from the Advisory Committee on Immunization Practices, which might have influenced who got vaccinated. Early divergence of Kaplan−Meier curves, while biologically plausible, can be a sign of unmeasured confounding (although later convergence of these curves is incompatible with ongoing influence of time-invariant confounders). However, the absence of association with a negative control outcome, the lack of difference in clinical trajectories in the year before vaccination, the similarities in preventive healthcare use during follow-up and the similar risk of non-cardiovascular death provide support for an absence of overt unmeasured confounders. Furthermore, confounding by secular trends is unlikely given the analysis comparing TDaP vaccine recipients in the two time windows.

In summary, receiving the recombinant shingles vaccine is associated with a reduced risk of cardiovascular events compared to the live attenuated vaccine. Given the global burden of cardiovascular disease, these findings, if confirmed in clinical trials and mechanistic studies, would have important implications for public health.

## Methods

### Study design and data source

We used EHR data from the TriNetX US Collaborative Network covering approximately 60 healthcare organizations (HCOs) and including data from more than 100 million patients^16–19^. Available data include demographics, diagnoses and medications. This study complies with Strengthening the Reporting of Observational Studies in Epidemiology (STROBE) guidelines and the Guidelines for Accurate and Transparent Health Estimates Reporting (GATHER) statement.

#### Acquisition of data, quality control and other procedures

Some descriptions of the TriNetX platform and its data quality procedures in this section are adapted from our previous studies that used the same network^6,16^. These studies provide more detail on how the network operates and how data flow from HCOs to the network. The available data modalities include demographics (encoded to HL7 v.3 administrative standards), diagnoses (International Classification of Diseases, 10th Revision, Clinical Modification (ICD-10-CM)), procedures (International Classification of Diseases, 10th Revision, Procedure Coding System (ICD-10-PCS) or Current Procedural Terminology (CPT)) and measurements (Logical Observation Identifiers Names and Codes (LOINC)). A typical HCO contributes approximately 9 years of historical data, occasionally extending to 15 years.

#### Mortality data

Mortality is ascertained from the EHR, which reliably records in-hospital deaths and captures some deaths occurring outside hospital; for a subset of patients, coverage of out-of-hospital deaths is improved by linkage to third-party sources (the Social Security Administration, private obituary records and private claims data).

### Ethics

TriNetX’s networks are compliant with the Health Insurance Portability and Accountability Act (HIPAA), the US federal law that protects the privacy and security of healthcare data. TriNetX is certified to the ISO 27001:2013 standard. All data accessible through the platform are deidentified in accordance with the HIPAA Privacy Rule (§164.514(b)(1) and §164.514(a)), with deidentification certified by a qualified statistical expert. On the basis of this expert determination, TriNetX is exempt from institutional review board (IRB) oversight. Participating HCOs supply data under Business Associate Agreements and warrant that they hold the rights and approvals required to do so, on condition that the contributing organizations remain unidentified and that the data are used solely for research. Keeping the identity of participating HCOs from the researchers using the data also contributes to complying with legal frameworks and ethical guidelines guarding against data reidentification. Because this study used only deidentified data, IRB approval and informed consent were not required, and no IRB protocol number applies.

### Assumptions for causal inference

Our study design can be formalized within an instrumental-variable framework, in which vaccination timing serves as an instrument for vaccine type. The five identifying assumptions of this framework for causal inference, together with the supporting evidence we provide, are as follows:

#### Relevance (assumption 1)

Compliance with the instrument was very high: 98.6% of individuals in the April−September 2017 cohort received the live shingles vaccine, and 93.5% of those in the April−September 2018 cohort received the recombinant shingles vaccine (Fig. 1a). Under intention-to-treat reasoning, this imperfect compliance dilutes the treatment contrast and biases the estimated associations toward the null rather than producing spurious associations^20^.

#### Exchangeability (assumption 2)

Although differences in age between cohorts were present before matching, they likely result from gradual drift in the demographics of people receiving the shingles vaccine. Indeed, there has been a gradual increase in the age of people being vaccinated against shingles, and this preceded the transition from the live to the recombinant shingles vaccine.^21^ This supports the assumption of conditional exchangeability.

After 1:1 propensity score matching, all covariates achieved standardized mean differences ≤0.1 (Supplementary Table 1). The matched cohorts entering the primary analysis are, therefore, well balanced on all observed sociodemographic, comorbidity and medication characteristics, supporting the observed component of conditional exchangeability.

Re-running propensity score matching with baseline covariates captured over different time windows (1, 2, 5 and 10 years before vaccination) yielded an RMTL ratio of 0.94 across all four windows (Table 1). The primary association is, therefore, consistent across the specification of covariate capture, indicating that it is not driven by cumulative state matching that could mask differential pre-vaccination trajectories.

The two cohorts behaved similarly in their preventive healthcare use before vaccination in terms of preventive health indicators. Similarly, the primary cardiovascular composite endpoint itself was null in the year before vaccination (RMTL ratio = 0.99, 95% CI: 0.92−1.06).

Although the above evidence supports exchangeability on observed dimensions, the influence of several unmeasured factors cannot be ruled out. Propensity score matching can only achieve balance of measured covariates, and the analysis of secular trends comparing TDaP vaccine recipients does not establish exchangeability between shingles vaccine cohorts. Socioeconomic status (education, income, occupation, neighborhood deprivation) is not captured in EHR data, and lifestyle factors (smoking, diet, alcohol use) are only indirectly and imperfectly captured through related clinical variables (obesity, diabetes, substance use disorder, etc.). If they systematically differ between cohorts after matching, such unmeasured confounders may violate the exchangeability assumption.

#### Exclusion restriction (assumption 3)

Comparing TDaP recipients vaccinated in the same calendar windows (April−September 2017 versus April−September 2018) showed no period effect on cardiovascular outcomes (RMTL ratio = 1.01, 95% CI: 0.99−1.03; Table 1). Because TDaP underwent no formulation change between these years, this rules out broad calendar time trends in cardiovascular diagnostic practice and intensity, healthcare delivery or background risk as alternative explanations.

The prespecified composite negative control outcome also supports the exclusion restriction. Because these conditions almost always require healthcare contact but have no suspected link to vaccination, a difference between cohorts would have indicated alternative pathways from vaccination timing to outcomes through generalized differences in diagnostic intensity or healthcare engagement; its null result, therefore, argues against such pathways. Likewise, cohorts were similar in their preventive healthcare use during the follow-up.

Restricting follow-up to before March 2020 yielded the same results. This rules out the possibility that our findings can be solely explained by pandemic-related changes in cardiovascular care seeking, which would have hit the two cohorts at different stages of follow-up because they were vaccinated 1 year apart.

#### Monotonicity (assumption 4)

A defier in this study would be an individual who would have selected the recombinant shingles vaccine in the April−September 2017 cohort if it had been available but actively refused it in the April−September 2018 cohort. The 6.5% of the April−September 2018 cohort who received the live shingles vaccine despite the recombinant vaccine availability are more likely to be ‘never-takers’ (individuals with a consistent preference for the live vaccine (for example, owing to single-dose preference or lower reactogenicity)) rather than defiers, because the same preferences would have led them to receive the live vaccine in 2017 as well. Defier behavior requires the implausible preference pattern of selecting the recombinant vaccine specifically when it is unavailable but rejecting it once available, for which no realistic clinical or behavioral mechanism exists. Monotonicity is, therefore, plausible by design.

#### Stable unit treatment value assumption (assumption 5)

Cardiovascular outcomes are individual-level events that do not transmit between people, so vaccinating one individual does not affect another’s cardiovascular risk. Both vaccines are well-defined biological products: the live attenuated vaccine and the recombinant vaccine are both FDA approved with consistent manufacturing during the study period, and no reformulations or sub-versions occurred within the cohort windows.

### Cohorts and exposures

Cohorts included all individuals who received their first shingles vaccine dose at the age of 60 or older between 1 April and 30 September 2018 (primary cohort) and between 1 April and 30 September 2017 (comparator cohort).

Participants were excluded if they had a diagnosis of hematological malignancy or immune deficiency recorded on or before their first singles vaccination, as these are relative contraindications for different vaccine types that would break the assumption behind the natural experiment:

- Malignant neoplasms of lymphoid, hematopoietic and related tissue (C81−C96)
- Monocytic leukemia (C93)
- Leukemia of unspecified cell type (C95)
- Lymphoid leukemia (C91)
- Other unspecified malignant neoplasms of lymphoid, hematopoietic and related tissue (C96)
- Myeloid leukemia (C92)
- Other leukemia of specified cell type (C94)
- Certain disorders involving the immune mechanism (D80−D89)
- HIV disease (B20)

For the same reason, we also excluded participants receiving treatments that affect their immune system within 1 year before the first singles vaccination, including:

- Immunosuppressants (Anatomical Therapeutic Chemical (ATC) code L04)
- Corticosteroids (ATC code R01AD)
- Chemotherapy (TriNetX-curated codelist 1002)
- Radiation oncology treatment (CPT code 1010843)

### Covariates

Evidence from the National Health Interview Survey^21^ indicates a gradual change in the demographic characteristics of individuals receiving shingles vaccination over time, with increasing coverage among older (≥70 years) compared to younger (60−69 years) adults preceding the transition from live to recombinant vaccines. If unadjusted for, these drifts in the vaccinated population characteristics would affect our effect estimates when comparing people vaccinated in two different calendar periods. Cohorts were, therefore, matched for 83 covariates, including sociodemographic factors, diagnoses, history of herpes infection, history of influenza vaccination and cardiovascular medications. All covariates (with ICD-10 codes for comorbidities) are listed in Supplementary Table 1. Covariates were selected as follows. All available sociodemographic factors were selected. These include age, sex (as recorded in the individual’s EHR), ethnicity, race and marital status. Age is reported as mean and standard deviation but was matched using 2-year bins (60−61, 62−63, …) up to age 95; those 95 and older were grouped together. This provides tighter control on age than using it as a continuous variable.

All broad ICD-10 categories of comorbidities were then included to balance comorbidity profiles between cohorts and because an indirect link with cardiovascular outcomes can be posited for most comorbidity profiles. Some broad ICD-10 categories were further broken down into their most prevalent constituents. This includes ‘Neoplasms’ (ICD-10 codes C00−D49), which was deemed too heterogeneous (because it includes both benign and malignant neoplasms); cardiovascular diseases (I00−I99); psychiatric disorders (F10−F59); and endocrine, nutritional and metabolic disorders (E00−E89), because they were deemed too heterogeneous and because they contain specific risk factors for cardiovascular outcomes, such as overweight and obesity, diabetes and thyroid disorders. In addition, prior herpes infections (both herpes simplex and herpes zoster) were included as covariates. Some factors affecting health and healthcare use (ICD-10 Z codes) were also included when they differed substantially between unmatched cohorts. Finally, to capture proxies of vaccine hesitancy, history of influenza vaccination (recommended every year for all adults in the United States) was included.

Adjustment for these covariates seeks to achieve conditional exchangeability of the cohorts. Notably, it cannot achieve exchangeability if there are unmeasured confounders.

### Outcomes

The primary outcome was diagnostic incidence of a composite cardiovascular endpoint comprising ischemic heart disease (ICD-10 codes I20−25), ischemic stroke (ICD-10 code I63) and heart failure (ICD-10 code I50) during a follow-up from 1 day to 7 years after vaccination. These were selected as they were found to be associated with the recombinant shingles vaccine in prior observation studies^2–4^ and are often combined in clinical trials of cardiovascular interventions^22^.

Because after 1:1 matching both cohorts are restricted to individuals who had a match in the other cohort, the estimand is best interpreted as the average treatment effect in the overlap population, in an intention-to-treat approach where treatment allocation depends on vaccination timing.

Secondary outcomes included atrial fibrillation (ICD-10 code I48); myocarditis (frequently misdiagnosed as ischemic heart disease; ICD-10 codes I09.0, I01.2, I40, I41, I51.4, B33.22 and D86.85); STEMI (ICD-10 codes I21.0−3), which is less likely to be a misdiagnosed myocarditis; peripheral arterial disease (to test for associations with atherosclerosis beyond the heart; ICD-10 codes I74.2 and I74.3); transient ischemic attack (ICD-10 codes G45.0, G45.1, G45.2, G45.3 and G45.9); hemorrhagic stroke (ICD-10 codes I60, I61 and I62); herpes zoster (shingles) infection (ICD-10 code B02); the composite of the primary endpoint or death; and a composite negative control outcome of any acutely painful condition as in our previous study^6^.

We also report each component of the composite primary endpoint separately. Although one component (for example, heart failure) could be a complication of another (for example, ischemic heart disease) occurring in the same follow-up, this could not be ascertained in the data.

### Definition of negative control outcome

Negative control outcomes are not typically associated with the primary outcome of interest and almost invariably require medical attention. We used a negative control composite outcome as defined in a previous publication^6^, as follows:

- Acute pancreatitis (ICD-10 code K85)
- Appendicitis (K35)
- Acute cholecystitis (K81.0)
- Adhesive capsulitis of the shoulder (M75.0)
- Trigeminal neuralgia (G50.0)

The negative control outcome was then defined as the composite outcome of a first diagnosis of any of these outcomes.

### Statistical analyses

Propensity score matching at a 1:1 ratio with a calliper of 0.1 was used to match cohorts on covariates. Characteristics with a standardized mean difference between cohorts of less than 0.1 were considered well matched^23^. The propensity score was calculated using a logistic regression (implemented by the function LogisticRegression of the scikit-learn package in Python 3.7) including each of the covariates mentioned above. To eliminate the influence of ordering of records, the record order in the covariate matrix was randomized before matching. The matching itself was performed using numpy 1.21.5 in Python 3.7.

The cumulative incidence of each outcome was estimated using the Kaplan−Meier estimator. Because we anticipated the proportional hazard assumption to be violated with such long follow-ups, differences between cohorts were summarized with the RMTL ratio via the survRM2 package (v.1.0.4)^24–26^. The RMTL ratio represents how much more time, on average, an individual has lived with the outcome during the follow-up period^27,28^. CIs were estimated using a parametric approach as defined in the survRM2 package in R^24^.

The *E* value was calculated to ascertain the sensitivity of the primary outcome to unmeasured confounders (using EValue package v.4.1.4). Given the absence of formulation of the *E* value for RMTL ratios, we used the *E* value formula for relative risks given that ratios of cumulative incidence can be interpreted as approximations of RMTL ratios: if the cumulative incidence functions are proportional to each other between cohorts (*F*_1_(*t*) = *R* × *F*_0_(*t*)), the RMTL ratio is equal to the proportionality constant (*R*). The *E* value represents how strongly an unmeasured confounder would need to be associated with both the exposure and the outcome, after adjusting for all measured confounders, to have a chance to explain away the association (that is, in a worst-case scenario where the prevalence of the unmeasured confounder maximizes its bias).

We calculated the difference in cumulative incidence as a function of time of follow-up by subtraction of the Kaplan−Meier curves. In addition, we calculated piecewise constant hazard ratios for the first half (1 day−3.5 years) and the second half (3.5−7 years) of follow-up.

Moderation by sex was tested using a permutation test, with 1,000 permutations as follows. The RMTL ratios between those vaccinated in 2018 and those vaccinated in 2017 were first calculated independently for men and women, and their difference was recorded. In each permutation, individuals were then randomly allocated to two groups of the same size as the initial ‘women’ and ‘men’ groups, and the analysis was repeated within these groups, thus leading to the calculation of RMTL ratios. The difference in absolute value between these RMTL ratios was recorded for each permutation, generating a distribution of 1,000 differences in RMTL ratios under the null hypothesis. The *P* value for the permutation test was calculated as follows: *P* = (1 + *n*_>_) / (1 + *n*), where *n* = 1,000 is the number of permutations and *n*_>_ is the number of permutations for which the difference in RMTL ratios was greater (in absolute value) than that observed in the non-permuted dataset.

Because we used EHRs with coded health events, if an event was not present it was considered absent. Missing data for sex, race and ethnicity were assigned their own category, which was included in the propensity score matching, so that the matched cohorts had approximately equal numbers of patients with unknown sex, race and ethnicity.

All analyses were performed in R (v.4.4.3) unless otherwise stated. Significance for all tests was set at two-sided *P* < 0.05.

### Secondary analysis

Analyses were repeated after (1) stratification by sex; (2) limiting follow-up to the first 518 days so that it occurred entirely before the COVID-19 pandemic; (3) using narrower ascertainment windows for baseline covariates (1, 2, 5 and 10 years before vaccination) while also adjusting for the number of healthcare encounters during these time windows, to assess whether matching on cumulative state covariates might mask differences in more recent pre-vaccination clinical trajectories; and (4) aligning follow-up times at the cohort level (by trimming follow-up to the shorter follow-up duration between randomly selected pairs of individuals) as in our previous study^6^ to assess sensitivity to cohort attrition and informative censoring.

To account for competing risks by non-cardiovascular death, we also estimated the cumulative incidence function (CIF) of the composite cardiovascular endpoint using the Aalen−Johansen estimator (cmprsk package v.2.2.12). Equality of CIFs between cohorts was tested with Gray’s non-parametric test (cmprsk package v.2.2.12). Sub-distribution hazard ratios were obtained from a Fine−Gray model^29^ fitted via the weighted Cox reformulation of Geskus with cluster-robust variance (‘survival’ package v.3.8.6); the proportional hazards assumption was assessed using scaled Schoenfeld residuals (‘survival’ package v.3.8.6), and, when violated, we fitted a time-segmented Fine−Gray model across four follow-up windows (0−1, 1−3, 3−5 and 5−7 years). For consistency with the primary analysis, we also report the Aalen−Johansen-based restricted mean time lost (AJ-RMTL) ratio by direct integration of the Aalen−Johansen CIF curves.

To assess potential secular trends in cardiovascular event incidence, such as changes in diagnostic practices or prevention over time, we compared the risk of the primary outcome among individuals who received a TDaP vaccine between April and September 2017 with those vaccinated during the same months in 2018. TDaP was selected because it is recommended for adults at 10-year intervals, unlike influenza vaccination, which is recommended annually. This comparison is subject to the same underlying secular trends as our primary analysis but does not involve the shingles vaccine transition. The TDaP cohorts were approximately 10 times larger than those in the primary analysis, providing substantially greater statistical power to detect any influence of temporal trends on the outcome. Besides the differences in cohort definitions, this analysis was otherwise conducted exactly as the primary analysis.

To assess whether people vaccinated against shingles in April−September 2018 had similar preventive healthcare use before vaccination as those vaccinated in April−September 2017, we compared preventive health indicators in the year before vaccination. We restricted the primary cohorts to individuals with a healthcare encounter 1−2 years before vaccination—that is, between April 2015 and March 2016 for those vaccinated in April−September 2017 and between April 2016 and March 2017 for those vaccinated in April−September 2018. We then rematched the cohorts on the same covariates as above but measured only before this earlier healthcare encounter, to avoid matching on healthcare use during the year immediately preceding vaccination.

We examined several indicators of preventive healthcare use, including influenza and pneumococcal vaccination; prescriptions for statins, antihypertensives and antidiabetics; and breast and bowel cancer screening. However, some of these may vary across calendar periods for reasons unrelated to shingles vaccination timing, limiting their value for this comparison. To identify indicators unaffected by such secular trends, we applied the same approach as above, comparing their frequency in the year before TDaP vaccination received in April−September 2018 versus April−September 2017. Only influenza and pneumococcal vaccination showed no systematic difference between the two periods, so we used these as indicators, together with a general code for preventive medicine services (CPT codes 1013829 and 1013859). Using the same approach, we also compared the risk of the primary cardiovascular endpoint in the year before shingles vaccination between the 2017 and 2018 cohorts to assess whether they were on similar cardiovascular health trajectories before vaccination.

Similarly, we assessed preventive healthcare use between cohorts during the follow-up. We considered the same preventive healthcare indicators as above and selected those that showed no secular trends (as detected by comparing cohorts who received TDaP vaccines). This led to the selection of bowel cancer screening (ICD-10-CM Z12.11) and antihypertensive prescription (VA CV100 or CV200 or CV800 or CV701 or CV805), together with a general code for preventive medicine services (CPT codes 1013829 and 1013859).

We also compared the recombinant shingles vaccine with two additional vaccines (namely, TDaP and influenza) given in April−September 2018.

### Reporting summary

Further information on research design is available in the Nature Portfolio Reporting Summary linked to this article.

## Online content

Any methods, additional references, Nature Portfolio reporting summaries, source data, extended data, supplementary information, acknowledgements, peer review information; details of author contributions and competing interests; and statements of data and code availability are available at https://doi.org/10.1038/s41591-026-04606-0.

## Supplementary information

Supplementary Information Supplementary Tables 1−8 and their legends.

Reporting Summary

Peer Review File

## Data Availability

The TriNetX system returned the results of these analyses as .csv files, which we downloaded and archived. Aggregate data, as presented in this article, can be freely accessed at https://osf.io/f25w6. The data used for this article were acquired from TriNetX. This study had no special privileges. Inclusion criteria specified in Methods would allow other researchers to identify similar cohorts of patients as we used here for these analyses; however, TriNetX is a live platform with new data being added daily, so exact counts will vary. To gain access to the data, a request can be made to TriNetX (join@trinetx.com), but costs might be incurred, and a data-sharing agreement would be necessary.

## Code availability

The code used to generate the results of this analysis can be freely accessed at https://osf.io/f25w6.
