# Supplementary material for: Recombinant shingles vaccination and the risk of cardiovascular events
Source: Nat Med. 2026 Aug 26;32(10):3612–6. doi: 10.1038/s41591-026-04606-0 (PMC13668725; doi:10.1038/s41591-026-04606-0)
Supplement: Supplementary file 1 [file 41591_2026_4606_MOESM1_ESM.pdf]

nature medicine

Supplementary information

https://doi.org/10.1038/s41591-026-04606-0

Recombinant shingles vaccination and the risk of cardiovascular events

In the format provided by the authors and unedited

Supplementary Material

Supplementary Table 1. Baseline characteristics before and after matching. ICD-10-CM codes are provided in brackets. SMD = Standardised mean difference.

	Before matching			After matching		
	**Vaccinated in Apr-Sep 2018**	**Vaccinated in Apr-Sep 2017**	**SMD**	**Vaccinated in Apr-Sep 2018**	**Vaccinated in Apr-Sep 2017**	**SMD**
**Number**	91854	37364	-	36460	36460	-
**SOCIODEMOGRAPHICS**						
Age; mean (SD); y	69.27 (6.36)	65.48 (5.63)	0.63	65.54 (5.49)	65.60 (5.65)	0.01
Sex; n (%)						
Female	51049 (55.58)	20477 (54.80)	0.016	19906 (54.60)	19977 (54.79)	0.0039
Male	40323 (43.90)	16855 (45.11)	0.024	16516 (45.30)	16451 (45.12)	0.0036
Other/Unknown	7143 (7.78)	3101 (8.30)	0.019	3043 (8.35)	3007 (8.25)	0.0036
Race; n (%)						
White	74775 (81.41)	28452 (76.15)	0.13	28278 (77.56)	27992 (76.78)	0.019
Black or African American	5487 (5.97)	4390 (11.75)	0.2	3828 (10.50)	4021 (11.03)	0.017
Asian	5045 (5.49)	1750 (4.68)	0.037	1763 (4.83)	1731 (4.75)	0.0041
American Indian or Alaska Native	188 (0.20)	110 (0.29)	0.018	112 (0.31)	107 (0.29)	0.0025
Native Hawaiian or Other Pacific Islander	434 (0.47)	225 (0.60)	0.018	209 (0.57)	218 (0.60)	0.0032
Other	1664 (1.81)	737 (1.97)	0.012	683 (1.87)	720 (1.98)	0.0074
Unknown	4261 (4.64)	1700 (4.55)	0.0043	1587 (4.35)	1671 (4.58)	0.011
Ethnicity; n (%)						
Hispanic or Latino	2903 (3.16)	1796 (4.81)	0.084	1634 (4.48)	1693 (4.64)	0.0078
Not Hispanic or Latino	81808 (89.06)	32467 (86.89)	0.067	31783 (87.17)	31760 (87.11)	0.0019
Unknown	7143 (7.78)	3101 (8.30)	0.019	3043 (8.35)	3007 (8.25)	0.0036
Marital status; n (%)						
Married	29617 (32.24)	12844 (34.38)	0.045	12727 (34.91)	12498 (34.28)	0.013
Never married	5667 (6.17)	3390 (9.07)	0.11	3161 (8.67)	3160 (8.67)	9.70E-05
Divorced	4079 (4.44)	2195 (5.88)	0.065	2151 (5.90)	2106 (5.78)	0.0053
Domestic partner	343 (0.37)	137 (0.37)	0.0011	135 (0.37)	136 (0.37)	0.00045
**COMORBIDITIES [ICD-10 code]; n (%)**						
Certain infectious and parasitic diseases [A00-B99]	17516 (19.07)	6267 (16.77)	0.06	6112 (16.76)	6141 (16.84)	0.0021
Herpesviral [herpes simplex] infections [B00]	1427 (1.55)	466 (1.25)	0.026	444 (1.22)	458 (1.26)	0.0035
Zoster [herpes zoster] [B02]	2683 (2.92)	863 (2.31)	0.038	854 (2.34)	851 (2.33)	0.00054
Neoplasms [C00-D49]	27378 (29.81)	9209 (24.65)	0.12	8998 (24.68)	9058 (24.84)	0.0038
Skin cancer [C43-C44]	4658 (5.07)	1122 (3.00)	0.11	1083 (2.97)	1118 (3.07)	0.0056
In situ neoplasms [D00-D09]	1967 (2.14)	484 (1.29)	0.065	480 (1.32)	480 (1.32)	0
Benign neoplasms [D10-D36]	18355 (19.98)	6176 (16.53)	0.089	6024 (16.52)	6076 (16.66)	0.0038
Neoplasms of uncertain behavior [D37-D48]	6031 (6.57)	1586 (4.25)	0.1	1530 (4.20)	1570 (4.31)	0.0054
Haematological conditions [D50-D89]	14232 (15.49)	4640 (12.42)	0.089	4492 (12.32)	4546 (12.47)	0.0045

Disorders of thyroid gland [E00-E07]	14884 (16.20)	5054 (13.53)	0.075	4917 (13.49)	4975 (13.64)	0.0046
Iodine-deficiency disorder [E01]	903 (0.98)	403 (1.08)	0.0095	384 (1.05)	388 (1.06)	0.0011
Other hypothyroidism [E03]	11716 (12.76)	3865 (10.34)	0.075	3743 (10.27)	3819 (10.47)	0.0068
Other nontoxic goiter [E04]	3404 (3.71)	1188 (3.18)	0.029	1206 (3.31)	1154 (3.17)	0.0081
Thyrotoxicosis [hyperthyroidism] [E05]	1152 (1.25)	432 (1.16)	0.009	401 (1.10)	423 (1.16)	0.0057
Autoimmune thyroiditis [E06.3]	651 (0.71)	187 (0.50)	0.027	189 (0.52)	187 (0.51)	0.00077
Other disorders of thyroid [E07]	1897 (2.06)	660 (1.77)	0.022	607 (1.67)	651 (1.79)	0.0093
Diabetes mellitus [E08-E13]	15040 (16.37)	6300 (16.86)	0.013	6027 (16.53)	6092 (16.71)	0.0048
Deficiency of other B group vitamins [E53]	3300 (3.59)	955 (2.56)	0.06	926 (2.54)	946 (2.60)	0.0035
Vitamin D deficiency [E55]	14306 (15.57)	4808 (12.87)	0.078	4716 (12.94)	4730 (12.97)	0.0011
Overweight and obesity [E66]	12381 (13.48)	5088 (13.62)	0.004	4964 (13.62)	4949 (13.57)	0.0012
Metabolic disorders [E70-E88]	46405 (50.52)	17128 (45.84)	0.094	16799 (46.08)	16755 (45.95)	0.0024
Psychiatric comorbidities						
Substance use disorder [F10-F19]	6538 (7.12)	3270 (8.75)	0.06	3172 (8.70)	3140 (8.61)	0.0031
Mood disorder [F30-F39]	11589 (12.62)	4810 (12.87)	0.0077	4730 (12.97)	4658 (12.78)	0.0059
Anxiety disorder [F40-F48]	13437 (14.63)	4964 (13.29)	0.039	4918 (13.49)	4876 (13.37)	0.0034
Organic disorder [F50-F59]	3018 (3.29)	1037 (2.77)	0.03	994 (2.73)	1018 (2.79)	0.004
Neurological disorder [G00-G99]	31837 (34.66)	11880 (31.80)	0.061	11502 (31.55)	11613 (31.85)	0.0065
Ophthalmic disease [H00-H59]	18937 (20.62)	6497 (17.39)	0.082	6227 (17.08)	6354 (17.43)	0.0092
Ear/mastoid process disease [H60-H95]	17226 (18.75)	5393 (14.43)	0.12	5343 (14.65)	5327 (14.61)	0.0012
Diseases of the circulatory system [I00-I99]	48707 (53.03)	18847 (50.44)	0.052	18204 (49.93)	18333 (50.28)	0.0071
Essential (primary) hypertension [I10]	39904 (43.44)	15638 (41.85)	0.032	15091 (41.39)	15176 (41.62)	0.0047
Hypertensive diseases [I10-I15]	40147 (43.71)	15759 (42.18)	0.031	15216 (41.73)	15292 (41.94)	0.0042
Ischaemic heart disease [I20-I25]	10636 (11.58)	3599 (9.63)	0.063	3520 (9.65)	3522 (9.66)	0.00019
Other forms of heart disease [I30-I52]	17454 (19.00)	5812 (15.55)	0.091	5539 (15.19)	5704 (15.64)	0.013
Chronic rheumatic heart diseases [I05-I09]	2280 (2.48)	591 (1.58)	0.064	574 (1.57)	583 (1.60)	0.002
Cerebrovascular diseases [I60-I69]	5907 (6.43)	1949 (5.22)	0.052	1841 (5.05)	1906 (5.23)	0.0081
Cerebral infarction [I63]	2162 (2.35)	828 (2.22)	0.0092	786 (2.16)	804 (2.21)	0.0034
Atherosclerosis [I70]	2998 (3.26)	850 (2.27)	0.06	809 (2.22)	835 (2.29)	0.0048
Other peripheral vascular diseases [I73]	3072 (3.34)	996 (2.67)	0.04	950 (2.61)	977 (2.68)	0.0046
Other disorders of arteries/arterioles [I77]	2153 (2.34)	622 (1.67)	0.048	638 (1.75)	612 (1.68)	0.0055
Disease of veins/lymphatics [I80-I89]	6033 (6.57)	2042 (5.46)	0.046	1948 (5.34)	2004 (5.50)	0.0068
Unspecified disorder of circulatory system [I95-I99]	3310 (3.60)	981 (2.63)	0.056	959 (2.63)	962 (2.64)	0.00051
Respiratory disease [J00-J99]	31723 (34.54)	11617 (31.09)	0.073	11382 (31.22)	11386 (31.23)	0.00024
Diseases of the digestive system [K00-K95]	37780 (41.13)	14035 (37.56)	0.073	13609 (37.33)	13736 (37.67)	0.0072
Dermatological disease [L00-L99]	27484 (29.92)	9504 (25.44)	0.1	9323 (25.57)	9345 (25.63)	0.0014
Musculoskeletal disease [M00-M99]	49713 (54.12)	18982 (50.80)	0.066	18465 (50.65)	18534 (50.83)	0.0038
Rheumatoid arthritis with rheumatoid factor [M05]	324 (0.35)	64 (0.17)	0.035	65 (0.18)	64 (0.18)	0.00065
Other rheumatoid arthritis [M06]	1183 (1.29)	358 (0.96)	0.031	359 (0.98)	350 (0.96)	0.0025
Wegener's granulomatosis [M31.3]	24 (0.03)	10 (0.03)	0.0004	10 (0.03)	10 (0.03)	0
Sjögren syndrome [M35.0]	407 (0.44)	95 (0.25)	0.03	88 (0.24)	95 (0.26)	0.0038
Dermatopolymyositis [M33]	66 (0.072)	15 (0.04)	0.013	18 (0.049)	15 (0.041)	0.0039

Genitourinary disease [N00-N99]	35712 (38.88)	12899 (34.52)	0.09	12570 (34.48)	12627 (34.63)	0.0033
Injury/poisoning [S00-T88]	24728 (26.92)	8991 (24.06)	0.066	8783 (24.09)	8797 (24.13)	9.00E-04
Falls [W00-W19]	3989 (4.34)	1129 (3.02)	0.07	1139 (3.12)	1113 (3.05)	0.0041
**FACTORS AFFECTING HEALTH**						
Long-term drug therapy [Z79]	17421 (18.97)	5142 (13.76)	0.14	5136 (14.09)	5084 (13.94)	0.0041
History of nicotine dependence [Z87.891]	8988 (9.79)	2371 (6.35)	0.13	2322 (6.37)	2348 (6.44)	0.0029
History of coded normal general examination [Z00.00]	31913 (34.74)	11673 (31.24)	0.075	11507 (31.56)	11453 (31.41)	0.0032
History of cancer screening [Z12]	32819 (35.73)	12017 (32.16)	0.075	11936 (32.74)	11818 (32.41)	0.0069
Problems related to housing and economic circumstances [Z59]	173 (0.19)	109 (0.29)	0.021	92 (0.25)	103 (0.28)	0.0058
**MEDICATIONS / VACCINES; n (%)**						
Flu vaccination	51885 (56.49)	20430 (54.68)	0.036	20226 (55.47)	19988 (54.82)	0.013
Beta blockers/related	21281 (23.17)	7198 (19.27)	0.096	6970 (19.12)	7047 (19.33)	0.0054
Calcium channel blockers	15514 (16.89)	5455 (14.60)	0.063	5324 (14.60)	5304 (14.55)	0.0016
Antihypertensive combinations	95 (0.10)	24 (0.064)	0.014	25 (0.069)	24 (0.066)	0.0011
Antihypertensives; other	9713 (10.57)	3374 (9.03)	0.052	3268 (8.96)	3302 (9.06)	0.0033
ACE inhibitors	18257 (19.88)	7206 (19.29)	0.015	6972 (19.12)	7012 (19.23)	0.0028
Angiotensin II inhibitor	13014 (14.17)	3947 (10.56)	0.11	3932 (10.78)	3894 (10.68)	0.0034
Anticoagulants	12311 (13.40)	3938 (10.54)	0.088	3856 (10.58)	3852 (10.56)	0.00036
Other cardiovascular medications	54276 (59.09)	19137 (51.22)	0.16	18721 (51.35)	18773 (51.49)	0.0029

***Beta blockers/related:** timolol, atenolol, acebutolol, betaxolol, bisoprolol, carvedilol, nebivolol, esmolol, labetalol, metoprolol, nadolol, pindolol, propranolol, sotalol; **Calcium channel blockers:** verapamil, amlodipine, clevidipine, levamlodipine, isradipine, diltiazem, felodipine, nicardipine, nifedipine, nimodipine, nisoldipine; **Antihypertensive combinations:** bendroflumethiazide, sacubitril; **Antihypertensives (other):** trimethaphan, sildenafil, riociguat, macitentan, clonidine, metyrosine, sotatercept, aprocitentan, lofexidine, treprostinil, tadalafil, guanfacine, iloprost, guanabenz, hydralazine, mecamylamine, methyldopa, methyldopate, minoxidil, nitroprusside; **ACE inhibitors:** benazepril, captopril, lisinopril, moexipril, quinapril, ramipril, enalapril, enalaprilat, trandolapril, fosinopril, perindopril; **Angiotensin II inhibitors:** azilsartan, candesartan, olmesartan, losartan, valsartan, telmisartan, eprosartan, irbesartan; **Anticoagulants**: dabigatran etexilate, rivaroxaban, warfarin, desirudin, defibrotide, apixaban, argatroban, edoxaban, heparin (porcine), lepirudin, fondaparinux, heparin, bivalirudin, enoxaparin, dalteparin, tirofiban, and eptifibatide. **Other cardiovascular medications:** *Digitalis glycosides:* digoxin; *Alpha blockers/related:* alfuzosin, terazosin, doxazosin, silodosin, tamsulosin, prazosin; *Antianginals:* pentaerythritol, ranolazine, nitroglycerin, isosorbide, isosorbide dinitrate, amyl nitrite; *Antiarrhythmics:* bretylium, dronedarone, adenosine, disopyramide, ibutilide, flecainide, dofetilide, lidocaine, mexiletine, amiodarone, procainamide, propafenone, quinidine; *Antilipemic agents:* icosapent ethyl, lomitapide, colesevelam, alirocumab, evolocumab, sebelipase alfa, cerivastatin sodium, bempedoic acid, cholestyramine resin, evinacumab, inclisiran, colestipol, olezarsen, plozasiran, rosuvastatin, ezetimibe, simvastatin, fluvastatin, pravastatin, gemfibrozil, omega-3 acid ethyl esters, lovastatin, atorvastatin, pitavastatin, probucol, fenofibrate; *Peripheral vasodilators:* cyclandelate, iloprost, isoxsuprine, nylidrin, papaverine; *Sclerosing agents:* ethanolamine oleate, polidocanol, sodium morrhuate, sodium tetradecyl sulfate; *Thiazide/related diuretics:* chlorothiazide, chlorthalidone, hydrochlorothiazide, indapamide, metolazone; *Loop diuretics:* bumetanide, torsemide, furosemide, ethacrynate; *Carbonic anhydrase inhibitor diuretics:* acetazolamide, dichlorphenamide, methazolamide; *Potassium-sparing diuretics:* triamterene, finerenone, eplerenone, amiloride, spironolactone; *Other diuretics:* pamabrom, mannitol; *Direct renin inhibitor:* aliskiren; *Other cardiovascular agents:* droxidopa, tafamidis, ivabradine, selexipag, angiotensin II, cilostazol, vericiguat, mavacamten, acoramidis, atrasentan, treprostinil, ambrisentan, milrinone, indomethacin, midodrine, inamrinone, bosentan, pentoxifylline.

Supplementary Table 2. Sex-stratified analyses for individual components of the primary outcome and moderation by sex.

**Moderation by sex**	**P value (permutation test)***
Composite cardiovascular endpoint	0.53
Ischemic Heart Disease	0.59
Ischemic stroke	0.58
Heart Failure	0.13

* P>0.05 indicates the absence of significant moderation by sex.

Supplementary Table 3. Time-segmented Fine–Gray sub-distribution hazard ratios for the composite cardiovascular endpoint accounting for death as a competing risk.

**Interval**	**Fine-Gray sHR(95%CI)**	**P value**
0–1 year	0.85 (0.78–0.94)	0.0050
1–3 years	0.82 (0.77–0.88)	7.51×10^{-9}
3–5 years	1.01 (0.95–1.08)	0.76
5–7 years	1.04 (0.98–1.10)	0.22
0–7 years (whole follow-up)	0.94 (0.91–0.97)	< 0.00001

Supplementary Table 4. Results from the analysis with the follow-up restricted to before the COVID-19 pandemic (i.e. a 518 days follow-up).

**Outcome**	**RMTL ratio (95% CI)**	**P value**
Composite endpoint	0.89 (0.82–0.97)	0.0068
Ischaemic heart disease	0.88 (0.80–0.97)	0.0078
Heart failure	0.86 (0.75–0.99)	0.038
Ischaemic stroke	0.91 (0.75–1.11)	0.37

Supplementary Table 5. Follow-up time in person-years, number of composite cardiovascular endpoint cases, and number lost to follow-up (e.g., because they made no further contact with their healthcare provider or moved to a different area). These data are presented for each year of follow-up and for both the primary analysis and after alignment of the follow-ups.

Primary analysis	Year 1	Year 2	Year 3	Year 4	Year 5	Year 6	Year 7
**Vaccinated in Apr-Sep 2017**							
**Person-years**	31,206	29,625	28,006	26,392	24,707	22,691	20,448
**Events**	1,015	1,012	923	920	1,056	1,177	1,134
**Censorings**	549	562	787	644	807	953	1,173
**Vaccinated in Apr-Sep 2018**							
**Person-years**	31,288	29,705	28,185	26,519	24,509	22,235	19,180
**Events**	912	809	763	1,000	1,026	1,135	1,098
**Censorings**	609	852	667	908	1,048	1,391	3,098
**Aligned follow-up**	**Year 1**	**Year 2**	**Year 3**	**Year 4**	**Year 5**	**Year 6**	**Year 7**
**Vaccinated in Apr-Sep 2017**							
**Person-years**	30,455	27,444	24,612	21,829	18,875	15,753	12,242
**Events**	987	937	797	764	802	813	694
**Censorings**	2,028	2,063	1,945	2,114	2,204	2,424	3,422
**Vaccinated in Apr-Sep 2018**							
**Person-years**	30,455	27,444	24,612	21,829	18,875	15,753	12,242
**Events**	888	744	669	846	794	807	724
**Censorings**	2,127	2,256	2,073	2,032	2,212	2,430	3,392

Supplementary Table 6. Comparison between cohorts in terms of pre-vaccination preventive care indicators (selected not to show secular trends during those calendar periods) and cardiovascular endpoint.

Outcome in the year before vaccination	RMTL ratio (95% CI)	P value
Influenza vaccination	1.02 (0.99–1.05)	0.26
Pneumococcal vaccination	1.03 (0.94–1.12)	0.57
Preventive medicine services	1.02 (0.96–1.08)	0.58
Cardiovascular composite endpoint	0.99 (0.92–1.06)	0.72

Supplementary Table 7. Comparison between cohorts in terms of post-vaccination preventive care indicators (selected not to show secular trends during those calendar periods).

Outcome in the year before vaccination	RMTL ratio (95% CI)	P value
Bowel cancer screening	1.00 (0.97–1.02)	0.70
Anti-hypertensive prescription	1.00 (0.99–1.01)	0.90
Preventive medicine services	1.01 (0.98–1.04)	0.46

Supplementary Table 8. Summary results for the comparison between the recombinant shingles vaccine and two other commonly used vaccines.

	N	RMTL ratio (95% CI)	P-value
**Recombinant shingles vs Influenza vaccine**			
Cardiovascular composite endpoint	94,923	0.75 (0.74 – 0.77)	6.1×10^{-190}
Ischaemic heart disease	94,923	0.78 (0.76 – 0.759)	1.3×10^{-120}
Ischaemic stroke	94,923	0.72 (0.69 – 0.75)	1.3×10^{-56}
Heart failure	94,923	0.69 (0.67 – 0.70)	1.5×10^{-174}
**Recombinant shingles vs Tdap vaccine**			
Cardiovascular composite endpoint	39,087	0.82 (0.80 – 0.85)	7.4×10^{-34}
Ischaemic heart disease	39,087	0.86 (0.83 – 0.89)	4.8×10^{-18}
Ischaemic stroke	39,087	0.75 (0.70 – 0.80)	5.0×10^{-17}
Heart failure	39,087	0.77 (0.73 – 0.80)	1.6×10^{-30}
