# Supplementary material for: Recombinant shingles vaccination and the risk of cardiovascular events
Source: Nat Med. 2026 Aug 26;32(10):3612–6. doi: 10.1038/s41591-026-04606-0 (PMC13668725; doi:10.1038/s41591-026-04606-0)
Supplement: Supplementary file 2 [file 41591_2026_4606_MOESM2_ESM.pdf]

nature portfolio

Corresponding author(s): Maxime Taquet

Last updated by author(s): Jun 16, 2026

Reporting Summary

Nature Portfolio wishes to improve the reproducibility of the work that we publish. This form provides structure for consistency and transparency in reporting. For further information on Nature Portfolio policies, see our Editorial Policies and the Editorial Policy Checklist.

Statistics

For all statistical analyses, confirm that the following items are present in the figure legend, table legend, main text, or Methods section.

n/a	Confirmed	
☐	☒	The exact sample size (n) for each experimental group/condition, given as a discrete number and unit of measurement
☐	☒	A statement on whether measurements were taken from distinct samples or whether the same sample was measured repeatedly
☐	☒	The statistical test(s) used AND whether they are one- or two-sided<br>*Only common tests should be described solely by name; describe more complex techniques in the Methods section.*
☐	☒	A description of all covariates tested
☐	☒	A description of any assumptions or corrections, such as tests of normality and adjustment for multiple comparisons
☐	☒	A full description of the statistical parameters including central tendency (e.g. means) or other basic estimates (e.g. regression coefficient) AND variation (e.g. standard deviation) or associated estimates of uncertainty (e.g. confidence intervals)
☐	☒	For null hypothesis testing, the test statistic (e.g. F, t, r) with confidence intervals, effect sizes, degrees of freedom and P value noted<br>*Give P values as exact values whenever suitable.*
☒	☐	For Bayesian analysis, information on the choice of priors and Markov chain Monte Carlo settings
☒	☐	For hierarchical and complex designs, identification of the appropriate level for tests and full reporting of outcomes
☒	☐	Estimates of effect sizes (e.g. Cohen's d, Pearson's r), indicating how they were calculated

Our web collection on statistics for biologists contains articles on many of the points above.

Software and code

Policy information about availability of computer code

Data collection

The data are stored onboard a TriNetX appliance – a physical server residing at the institution's data centre or a virtual hosted appliance. The TriNetX platform is a fleet of these appliances connected into a federated network able to broadcast queries to each appliance. Results are subsequently collected and aggregated.

Once the data are sent to the network, they are mapped to a standard and controlled set of clinical terminologies and undergo a data quality assessment including 'data cleaning' that rejects records which do not meet the TriNetX quality standards. HIPAA compliance of the clinical patient data is achieved using de-identification. Different data modalities are available in the network. They include demographics (coded to HL7 version 3 administrative standards), diagnoses (represented by ICD-10-CM codes), procedures (coded in ICD-10-PCS or CPT), and measurements (coded to LOINC).

The data from a typical HCO generally go back around 9 years, with some going back 15 years. The data are continuously updated. HCOs update their data at various times, with most refreshing every 1, 2, or 4 weeks.

Data quality assessment followed a standardised strategy wherein the data are reviewed for conformance (adherence to specified standards and formats), completeness (quantifying data presence or absence) and plausibility (believability of the data from a clinical perspective). There are pre-defined metrics for each of the above assessment categories. Results for these metrics are visualised and reviewed for each new site that joins the network as well as on an ongoing basis. Any identified issue is communicated to the data provider and resolved before continuing data collection.

The basic formatting of contributed data is also checked (e.g. to ensure that dates are properly represented). Records are checked against a list of required fields (e.g., patient identifier) and rejects those records for which the required information is missing. Referential integrity

checking is done to ensure that data spanning multiple database tables can be successfully joined together. As the data are refreshed, changes in volume of data over time is monitored to ensure data validity. At least one non-demographic fact for each patient is required for them to be counted in the dataset. Patient records with only demographics information are discarded.

The software also undergoes quality control. The engineers testing the software are independent from the engineers developing it. Each test code is checked by two independent testing engineers. Each piece of software is tested extensively against a range of synthetic data (i.e. generated for the purpose of testing) for which the expected output is established independently. If the software fails to return this output, then the software is deemed to have failed the test and is examined and modified accordingly. For statistical software (including that used for propensity score matching, for Kaplan-Meier analysis, etc), an additional quality control step is implemented. Two independent codes are written in two different programming languages (R and python) and the statistical results are compared. If discrepancies are identified, then the codes are deemed to have failed the test and are examined and modified accordingly. All the code is reviewed independently by another engineer.

Propensity-score matching was achieved by calculating the propensity score with the scikit-learn package in Python 3.7 and using a nearest neighbour search with numpy 1.21.5 in Python 3.7.

The test strategy follows three levels of granularity:

1. Unit tests: These test specific blocks, or units, of code that perform specific actions (e.g. querying the database).
2. Integration tests: These ensure that different components are working together correctly.
3. End-to-end tests: These tests run the entire system and check the final output.

Data analysis

Data analysis includes all the processing of data from the life tables of matched cohorts and include Kaplan-Meier estimation, restricted mean time lost estimates, bootstrapping, permutation test and generation of figures. All these steps were conducted in R version 4.2.1. The assumption that the hazards were proportional was tested using the generalized Schoenfeld approach implemented in the cox.zph function of the survival package (version 3.8.6). The restricted mean time lost was calculated using R package survRM2 version 1.0.4. Bootstrapping and permutation tests were conducted using in-house R code that is publicly available. To account for the competing risk of non-cardiovascular death, we estimated cumulative incidence functions for the composite cardiovascular endpoint using the Aalen–Johansen estimator and compared cohorts using Gray's test (both implemented in cmprsk package v. 2.2.12). Subdistribution hazard ratios were estimated with Fine–Gray models using cluster-robust variance (package survival v. 3.8.6) and time-segmented models were fitted (package survival v. 3.8.6). For consistency with the primary analysis, we also report Aalen–Johansen-based restricted mean time lost ratios. The E-value for the primary restricted mean time lost ratio was calculated to assess the sensitivity of the result to unmeasured confounding, using the E-value formula for relative risks applied to the restricted mean time lost ratio (package EValue v. 4.1.4). The code used to generate the results of this analysis can be freely accessed at https://osf.io/f25w6

For manuscripts utilizing custom algorithms or software that are central to the research but not yet described in published literature, software must be made available to editors and reviewers. We strongly encourage code deposition in a community repository (e.g. GitHub). See the Nature Portfolio guidelines for submitting code & software for further information.

Data

Policy information about availability of data

All manuscripts must include a data availability statement. This statement should provide the following information, where applicable:

- Accession codes, unique identifiers, or web links for publicly available datasets
- A description of any restrictions on data availability
- For clinical datasets or third party data, please ensure that the statement adheres to our policy

The TriNetX system returned the results of these analyses as csv files, which we downloaded and archived. Aggregate data, as presented in this article, can be freely accessed at [url provided upon acceptance of the paper]. The data used for this article were acquired from TriNetX. This study had no special privileges. Inclusion criteria specified in the Methods would allow other researchers to identify similar cohorts of patients as we used here for these analyses; however, TriNetX is a live platform with new data being added daily so exact counts will vary. To gain access to the data, a request can be made to TriNetX (join@trinetx.com), but costs might be incurred, and a data sharing agreement would be necessary. Aggregate data, as presented in the article and the code used to generate the results can be freely accessed at https://osf.io/f25w6.

Research involving human participants, their data, or biological material

Policy information about studies with human participants or human data. See also policy information about sex, gender (identity/presentation), and sexual orientation and race, ethnicity and racism.

Reporting on sex and gender

Analysis stratified by sex was performed. The sex was as recorded in the individual's electronic health record.

Reporting on race, ethnicity, or other socially relevant groupings

Self-reported race and ethnicity in TriNetX come from the individual's electronic health record. TriNetX maps race to the following categories: Asian, American Indian or Alaskan Native, Black or African American, Native Hawaiian or Other, White, Unknown race. It maps ethnicity to the following categories: Hispanic or Latino, Not hispanic or latino, Unknown ethnicity.

Population characteristics

All these characteristics are reported in full in Supplementary Table 1

Recruitment

There was no recruitment as this was a real-world analysis based on electronic health records data. All individuals with a recorded shingles vaccine at the age of 60 or over, between 1 April 2017 and 30 September 2017 or between 1 April 2018 and 30 September 2018 and who did not have a diagnosis of haematological malignancy or immune deficiency recorded on or before their first singles vaccination, and did not received an immunosuppressant drug in the year before vaccination, were included.

Ethics oversight	De-identification data are formally attested as per Section §164.514(b)(1) of the HIPAA Privacy Rule, superseding TriNetX's waiver from the Western Institutional Review Board; no further ethical approval was thus needed.

Note that full information on the approval of the study protocol must also be provided in the manuscript.

Field-specific reporting

Please select the one below that is the best fit for your research. If you are not sure, read the appropriate sections before making your selection.

☒ Life sciences ☐ Behavioural & social sciences ☐ Ecological, evolutionary & environmental sciences

For a reference copy of the document with all sections, see nature.com/documents/nr-reporting-summary-flat.pdf

Life sciences study design

All studies must disclose on these points even when the disclosure is negative.

Sample size	This is a real-world data analysis which includes all individuals who received a shingles vaccine between 1 April 2017 and 30 September 2017 or between 1 April 2018 and 30 September 2018. No sample size calculation was therefore performed.
Data exclusions	Pre-established exclusion criteria included diagnosis of haematological malignancy or immune deficiency recorded on or before their first singles vaccination as these are relative contraindications for different vaccine types which would break the assumption behind the natural experiment. For the same reason, we also excluded participants receiving treatments that affect their immune system within one year before the first singles vaccination, including: immunosuppressants, corticosteroids, chemotherapy, and radiation oncology treatment.
Replication	Analyses were run once and findings were replicated among women and men.
Randomization	The influence of covariates was strongly limited by the use of a natural experiment created by the rapid transition from the use of live shingles vaccine to the use of recombinant shingles vaccine. Remaining covariates imbalance (e.g. due to drift in characteristics of the population being vaccinated) were further controlled for by propensity-score matching.
Blinding	Cohorts were created based on predefined inclusion and exclusion criteria. The outcome was ascertained based on routine electronic health records data and not by any investigator of this study. As a result, blinding was not necessary.

Reporting for specific materials, systems and methods

We require information from authors about some types of materials, experimental systems and methods used in many studies. Here, indicate whether each material, system or method listed is relevant to your study. If you are not sure if a list item applies to your research, read the appropriate section before selecting a response.

Materials & experimental systems

n/a	Involved in the study	
☒	☐	Antibodies
☒	☐	Eukaryotic cell lines
☒	☐	Palaeontology and archaeology
☒	☐	Animals and other organisms
☒	☐	Clinical data
☒	☐	Dual use research of concern
☒	☐	Plants

Methods

n/a	Involved in the study	
☒	☐	ChIP-seq
☒	☐	Flow cytometry
☒	☐	MRI-based neuroimaging

Plants

Seed stocks	N/A
Novel plant genotypes	N/A
Authentication	N/A
