# Supplementary material for: Recombinant shingles vaccination and the risk of cardiovascular events
Source: Nat Med. 2026 Aug 26;32(10):3612–6. doi: 10.1038/s41591-026-04606-0 (PMC13668725; doi:10.1038/s41591-026-04606-0)
Supplement: Supplementary file 3 [file 41591_2026_4606_MOESM3_ESM.pdf]

nature portfolio

Peer Review File

Recombinant shingles vaccination and the risk of cardiovascular events

Corresponding Author: Professor Maxime Taquet

Parts of this Peer Review File have been redacted as indicated to remove third-party material.

This file contains all reviewer reports in order by version, followed by all author rebuttals in order by version.

Version 0:

Reviewer comments:

Reviewer #1

(Remarks to the Author)
This study compared adults ≥60 vaccinated just before versus after the U.S. switch from live to recombinant shingles vaccine. The recombinant vaccine was associated with a 9% increase in cardiovascular diagnosis-free time over 7 years (RMTL ratio 0.91, 95% CI 0.88–0.95). It reduced risks of ischaemic heart disease and heart failure in both sexes, and ischaemic stroke in men, with additional reductions in atrial fibrillation but not myocarditis or peripheral arterial disease. Associations were strongest early and attenuated over time.

I believe that this contribution provides novel evidence on an important topic and is a contribution worth publishing. Clearly, if shingles vaccination truly has a substantial protective effect for cardio- and cerebrovascular disease, this would be important to know for physicians, patients, vaccination recommendation committees, and researchers studying the mechanisms that lead to such conditions. Having said this, I think it is absolutely critical that the manuscript undergoes major revisions (which I outline below), particularly with regards to the use of the term natural experiment, before it is considered for publication.

Major comments
1. The manuscript overstates the strength of the research design and uses overly strong causal language
The manuscript frames the study as a "natural experiment" in which vaccination timing "serves as a quasi-random assignment" and "eliminates healthy-vaccinee bias." It is my view that this characterization is not supported by the empirical evidence presented and should be changed.

Supplementary Table 1 reveals substantial pre-matching differences between the April–September 2018 and April–September 2017 cohorts, most strikingly in age (69.27 vs. 65.48 years; SMD 0.63), alongside imbalances across a range of demographic and clinical characteristics. Far from being minor residual discrepancies, they indicate that the two groups were materially different before any adjustment. Matching can improve balance on observed covariates, but it does not transform a fundamentally non-exchangeable before/after comparison into a natural experiment.

The quasi-random assignment assumption is further weakened by the specific context of the ZVL-to-RZV transition. This was not a neutral substitution: it followed updated recommendations from the Advisory Committee on Immunization Practices, which introduced preferential guidance for RZV and recommended vaccination of prior ZVL recipients.1 At the same time, RZV uptake was plausibly selective. The vaccine requires a two-dose schedule, carries greater reactogenicity, and was introduced under conditions of limited supply with considerable public attention. All of this raises the threshold of effort and tolerance required for uptake and makes a random-assignment approximation implausible.

This concern is compounded by important data limitations. The EHR data contain no direct information on education, income, occupation, or living conditions. The only available proxy for socioeconomic disadvantage ("problems related to housing and economic circumstances") is extremely rare (<0.3%) and is therefore unlikely to capture meaningful variation in health-seeking behavior. Given well-established associations between socioeconomic position, healthcare access, and preventive behavior, residual confounding along these dimensions remains a central and unresolved concern.

The design is, in my view, most accurately described as a matched observational comparison across two vaccine-market periods. While matching improves balance on measured variables, it does not establish exchangeability, particularly in the presence of unmeasured socioeconomic and healthcare-access factors. The current causal language accordingly goes further than the evidence allows. Statements that the study provides "causal rather than correlational evidence" or that the design "eliminates healthy-vaccinee bias" are not supported by the identification strategy. The supplement appropriately acknowledges residual confounding, but this caveat is absent from the main text, where it is most needed. A more defensible framing would describe the findings as associations consistent with a protective effect, rather than causal estimates.

It is also critical for the paper to report additional diagnostics on residual confounding. In particular, the authors should show if recent healthcare utilization, new diagnoses, or treatment changes showed different trends in the groups who received RZV and ZVL during the time before vaccination. This should be done by showing the time trajectories of preventive health indicators (influenza vaccination uptake, pneumococcal vaccination uptake, screening [e.g. colorectal cancer, skin cancer, breast cancer], new prescriptions of statins, anti-hypertensives or antidiabetic medications) and prior diagnoses of cardio-vascular diseases. Even if the two samples appear identical after matching on cumulative baseline indicators, this balance might mask different time trends prior to vaccination which would contribute to inter-group differences after vaccination. These diagnostics would not establish exchangeability but would offer more direct evidence on whether the matched groups were on comparable pre-vaccination health trajectories.

Finally, while similar "natural experiment" language has been used in the authors' prior work employing a comparable design,2 this previous language was, in my opinion, inappropriate, and the precedent does not justify the same language in the current study without substantial additional evidence corroborating the underlying assumptions. Specifically, previous studies have demonstrated that vaccination timing is associated with education, income, uptake of other vaccinations (e.g., influenza),3 in addition to ethnicity and self-rated health.4

2. Outcome definitions require clearer justification
The composite endpoint of ischemic heart disease, ischemic stroke, and heart failure is broadly motivated but requires more explicit justification at several points.

The omission of transient ischemic attack (TIA) is difficult to reconcile with the existing literature, which consistently treats stroke and TIA as related outcomes and reports elevated risk following herpes zoster.5-7 At a minimum, TIA should be included as a secondary outcome or its exclusion explicitly justified.

The inclusion of heart failure is less clearly supported. The association between herpes zoster and heart failure is weaker and partly bidirectional, with evidence pointing both to increased HF risk following zoster and to increased zoster risk among individuals with pre-existing HF.8,9 The manuscript should clarify whether heart failure here refers to incident disease, worsening of pre-existing disease, or HF arising as a complication of another cardiovascular event captured within the same follow-up window.
Finally, the exclusion of hemorrhagic stroke warrants explicit justification. Meta-analytic evidence identifies a significant association between herpes zoster and hemorrhagic stroke,10 meaning this outcome is not negligible a priori. In my view, hemorrhagic stroke should be included at least in secondary analyses.

3. The primary time-to-event analysis should account for competing risks
The analysis uses Kaplan–Meier methods in a cohort where death is a competing event of considerable magnitude. Because individuals who die cannot subsequently experience cardiovascular events, standard survival methods are misaligned with the clinical estimand of interest. In my view, a competing-risks framework (e.g., Aalen–Johansen estimators combined with cause-specific or Fine–Gray models) should be used in the primary analysis. The composite of the primary endpoint and death does not resolve this issue; it changes the estimand rather than addressing the underlying problem. Given the length of follow-up and the incomplete mortality capture inherent in TriNetX, this issue is particularly consequential.

4. Cohort attrition and healthcare engagement require explicit assessment
The manuscript does not report retention or loss to follow-up, despite prior work using the same data source documenting substantial attrition.2 In EHR-based analyses, continued observation depends on ongoing interaction with healthcare systems and is therefore likely informative rather than ignorable.

I believe that the authors should report retention over time, compare follow-up distributions between groups, and assess robustness to differential attrition. Without such diagnostics, long-term comparisons may reflect selection into continued observation rather than causal effects, a concern that appears particularly relevant given the attenuation of estimated effects beyond approximately 3.5 years.

This section also provides an opportunity to examine potential post-vaccination differences in health-seeking behavior. If RZV recipients differ systematically in healthcare engagement, this may be reflected in their subsequent utilization of other vaccines (e.g., pneumococcal or TDaP) and preventive services. Examining such patterns is, in my view, important and would provide indirect evidence on residual healthy-user bias as well as help contextualize the observed outcome trajectories.

5. Potential confounding from the COVID-19 pandemic
Outcome trajectories show a structural change at approximately 500 days of follow-up, corresponding to the onset of the COVID-19 pandemic. Pandemic-related declines in cardiovascular hospitalizations have been well documented11,12 and

likely reflect reduced care-seeking and altered diagnostic intensity rather than true changes in event incidence. The different patterns observed across outcomes (e.g., more abrupt for ischemic heart disease and heart failure, more gradual for stroke) introduce calendar-time confounding, as the two cohorts encounter the pandemic at different stages of follow-up. So, I would strongly recommend sensitivity analyses that restrict follow-up to before March 2020.

6. Estimand and external validity should be clarified
The matched analysis identifies an effect for the overlap population rather than for the full population of vaccine recipients. Given the substantial pre-matching differences documented in Supplementary Table 1, this distinction is consequential and should be stated explicitly. The manuscript should, in my opinion, define the estimand clearly and avoid generalizing findings to broader populations without appropriate qualification.

7. Effects on other outcomes should be documented
The fact that the authors observe an effect on cardiovascular outcomes in addition to the prevention of dementia raises questions about whether patients who received RZV are less likely than ZVL-receivers to be diagnosed with every kind of clinical condition in the follow-up period. Barring the possibility that RZV is a miracle cure for all diseases, such a set of results would imply there exist unobserved differences between patients who received RZV and ZVL not accounted for in the propensity-score matching algorithm.
To assuage these concerns, I believe it is critical that the authors show whether there are differences between RZV and ZVL for each condition in the Charlson Comorbidity Index or the, say, 20 most common diagnoses in their data.13 It would be very informative to show if the effect of HZ-vaccination on cardio-vascular conditions was specific or if similar associations appear with respect to other common diagnoses.

8. The manuscript should more clearly delineate what matching can and cannot achieve
Throughout the manuscript, propensity-score matching appears to be invoked as a remedy for concerns broader than covariate balance.14 Matching improves comparability on observed characteristics but does not address unmeasured confounding, differences in diagnostic intensity, or compositional changes in the vaccinated population across calendar periods. The manuscript would, in my view, benefit from a cleaner separation between the contribution of covariate adjustment and the requirements for causal identification, and should avoid implying that the former is sufficient for the latter.

Minor comments
The Discussion notes early divergence in outcome trajectories but moves quickly towards a biological interpretation. It would be important to acknowledge upfront that early divergence is also consistent with residual confounding or systematic differences in healthcare utilization between the two cohorts.

The TDaP comparison is a useful diagnostic tool for ruling out gross secular trends, but it does not establish exchangeability between the shingles vaccine cohorts. This distinction should be made more explicit.
There is meaningful heterogeneity in temporal trajectories across outcomes, particularly for ischemic heart disease, which shows an early decline followed by a reversal not observed in other outcomes. This pattern is not clinically intuitive and warrants more careful discussion. It may reflect changes in the composition of the risk set over time, or the well-documented sensitivity of IHD coding to healthcare utilization.

The manuscript would benefit from a clearer account of follow-up and censoring assumptions, particularly given that continued observation depends on ongoing interaction with the participating healthcare organizations.

Finally, there appears to be a coding discrepancy in the supplement, where both acute pancreatitis and acute cholecystitis are assigned ICD-10 code K81.0.

References
1. Dooling KL, Guo A, Patel M, et al. Recommendations of the Advisory Committee on Immunization Practices for Use of Herpes Zoster Vaccines. MMWR Morb Mortal Wkly Rep 2018;67:103–108. doi: http://dx.doi.org/10.15585/mmwr.mm6703a5.
2. Taquet M, Dercon Q, Todd JA, Harrison PJ. The recombinant shingles vaccine is associated with lower risk of dementia. Nat Med. 2024;30(10):2777-2781. doi:10.1038/s41591-024-03201-5
3. Kang AH, Kim JK, Ailshire J, Crimmins EM. Shingles Vaccine Uptake Among Older Adults: Identifying Early, Later, and Nonadopters. AJPM Focus. 2023;2(4):100143. doi:10.1016/j.focus.2023.100143
4. Vogelsang EM, Polonijo AN. Social Determinants of Shingles Vaccination in the United States. The Journals of Gerontology: Series B. 2022;77(2):407-412. doi:10.1093/geronb/gbab074
5. Zhang Y, Luo G, Huang Y, Yu Q, Wang L, Li K. Risk of Stroke/Transient Ischemic Attack or Myocardial Infarction with Herpes Zoster: A Systematic Review and Meta-Analysis. J Stroke Cerebrovasc Dis. 2017;26(8):1807-1816. doi:10.1016/j.jstrokecerebrovasdis.2017.04.013
6. Kwon SU, Yun SC, Kim MC, et al. Risk of stroke and transient ischaemic attack after herpes zoster. Clin Microbiol Infect. 2016;22(6):542-548. doi:10.1016/j.cmi.2016.03.003
7. Kumar N, Khera R, Pandey A, Garg N. Racial Differences in Outcomes after Acute Ischemic Stroke Hospitalization in the United States. J Stroke Cerebrovasc Dis. 2016;25(8):1970-1977. doi:10.1016/j.jstrokecerebrovasdis.2016.03.049
8. Seo HM, Cha MJ, Han JH, et al. Reciprocal relationship between herpes zoster and cardiovascular diseases: A nationwide population-based case–control study in Korea. The Journal of Dermatology. 2018;45(11):1312-1318. doi:10.1111/1346-8138.14597
9. Wu PH, Lin YT, Lin CY, Huang MY, Chang WC, Chang WP. A nationwide population-based cohort study to identify the correlation between heart failure and the subsequent risk of herpes zoster. BMC Infect Dis. 2015;15:17. doi:10.1186/s12879-015-0747-9

10. Lian Y, Zhu Y, Tang F, Yang B, Duan R. Herpes zoster and the risk of ischemic and hemorrhagic stroke: A systematic review and meta-analysis. PLoS One. 2017;12(2):e0171182. doi:10.1371/journal.pone.0171182
11. Bhatt AS, Moscone A, McElrath EE, et al. Fewer Hospitalizations for Acute Cardiovascular Conditions During the COVID-19 Pandemic. J Am Coll Cardiol. 2020;76(3):280-288. doi:10.1016/j.jacc.2020.05.038
12. Solomon MD, McNulty EJ, Rana JS, et al. The Covid-19 Pandemic and the Incidence of Acute Myocardial Infarction. N Engl J Med. 2020;383(7):691-693. doi:10.1056/NEJMc2015630
13. Charlson ME, Carrozzino D, Guidi J, Patierno C. Charlson Comorbidity Index: A Critical Review of Clinimetric Properties. Psychother Psychosom. 2022;91(1):8-35. doi:10.1159/000521288
14. Austin PC. An Introduction to Propensity Score Methods for Reducing the Effects of Confounding in Observational Studies. Multivariate Behav Res. 2011;46(3):399-424. doi:10.1080/00273171.2011.568786

Reviewer #2

(Remarks to the Author)
Summary
This study uses the switch from live to recombinant zoster vaccination in the US as a natural experiment to compare cardiovascular outcomes. In matched adults aged 60 and over, the recombinant vaccine was associated with a lower risk of the composite cardiovascular endpoint over 7 years. The association was clearest for IHD and HF, weaker for stroke, and attenuated over time. Secondary and control analyses were broadly consistent with the main findings.

Originality/significance
The design is more robust than prior vaccinated versus unvaccinated comparisons and addresses healthy vaccinee bias. However, as mentioned below, I am not sure what the quasi-randomised design is testing, since this is now a comparison of two vaccines rather than vaccination versus no vaccination. Is the question whether any zoster vaccine might prevent CVD, or whether recombinant vaccine is more protective than live vaccine? If it is the latter, the main justification given in the introduction feels somewhat tangential.

Data/methods
Large EHR dataset and extensive matching are strengths. The natural experiment is plausible but the assumptions is that people vaccinated just before and just after the transition should be comparable. Residual confounding, selection around the transition, and incomplete capture of care remain possible. I really appreciate the use of a negative control outcome (acutely painful conditions) to balance this.

Stats analyses
Appear appropriate.

Suggested improvements
1. The underlying biological hypothesis is not fully clear. On reading the first part of the main text I'm just not clear at what qasi-randomisation is getting at here as an exposure. Both vaccines prevent zoster, so it is not obvious why the recombinant vaccine would reduce CVD risk whereas the live vaccine would not. Please clarify the proposed mechanism and whether the expectation is a different effect between vaccine types or a general effect of zoster vaccine. Outline this more clearly in the introduction as well as revisiting it in the discussion. I would want to avoid the perception that the live vaccine is worthless in countries/areas where recombinant vaccine is not available.
2. Is it possible to provide stronger evidence that cohorts around the transition are similar?
3. Abstract - “Increased diagnosis free time” is technically correct but not the most natural way to think about the main reult. Most readers will find “reduced risk” or “lower incidence” easier to interpret, especially in an abstract. Just a suggestion.
4. I suggest tone down mechanistic and causal language. Notably, I think the dementia paper is more cautious about its limitations. It states plainly that causality cannot be demonstrated, whereas the language in this paper is a bit more causal e.g. “starts to provide causal evidence”.
5. As in the dementia paper, the authors suggest that these findings could lead to a more definitive RCT. I do not object to this idea, but I found it unclear what the proposed randomisation would actually be, given that randomisation to no vaccine would be ethically difficult.

Reviewer #3

(Remarks to the Author)
This is a nicely written paper describing a carefully conducted study that used a natural experiment created by the rapid transition from the live attenuated to the recombinant shingles vaccine in the USA, comparing the incidences of a composite cardiovascular endpoint among adults aged ≥60 years vaccinated immediately before versus immediately after this transition. The recombinant vaccine was associated with a 9% increase in cardiovascular diagnosis-free time over 7 years. Several approaches were used to reduce or assess confounding, and the results are rather robust. Negative controls are mentioned but not included in the paper, which they should be. Additionally, while it is likely that this study shows a decrease in risk of cardiovascular endpoints from the recombinant vaccine, it is also possible that the recombinant vaccine is not protective and in fact the live attenuated vaccine caused cardiovascular disease and the recombinant vaccine did not. This limitation of the study design should be acknowledged

Version 1:

Reviewer comments:

Reviewer #1

(Remarks to the Author)
I would like to thank the authors for their revisions. In my view, however, there are still several major concerns in the way in which the study is framed/described that in my opinion need to be addressed before publication. I will of course defer to the editorial team on these matters but would just like to take this opportunity to describe my point of view and reasoning for these opinions. Please find these below.

- Signs of selection bias.

I argued earlier that a main concern in this study is that outcomes may be driven by selection bias. Extended Data Figure 5 suggests some signs of this. In this figure, the authors present a comparison between Shingrix and a) influenza or b) TDaP vaccinators during the same time-period (April-September 2018). The authors present this comparison as additional evidence of the protective effect of Shingrix. But by their own stated logic, these comparisons are subject to the healthy-vaccinee bias that motivated their primary design in the first place. Notable, however, in these comparisons is that the "protective effect" roughly doubles in magnitude compared to the primary analysis (which attempts to control for vaccine selection). This is exactly what you would expect if selection bias was driving or inflating the estimates. Rather than reinforcing the analyses, these secondary comparisons may actually illustrate the magnitude of confounding that the primary design needs to overcome. I think the authors need to discuss this figure and what it may reveal about healthy vaccinee bias more thoroughly.

- E-value

To properly test whether outcomes are driven by selection bias needs a causal research design, which this study does not offer. In its place, I would recommend the study reports the E-value (VanderWeele and Ding 2017), which quantifies how strong an unmeasured confounder would need to be to fully explain an observed association. Reporting the E-value would provide needed transparency for an observational study like this (and is recommended by STROBE). Applied to the primary RMTL of 0.91 (95% CI 0.88–0.95), a rough calculation yields an E-value of approximately 1.43 for the point estimate and 1.29 for the confidence interval bound closest to the null. This is a little concerning (E-values <2 are usually seen as fragile, 2-5 as moderate, and >5 as large). An unmeasured confounder associated with both treatment assignment and the outcome by risk ratios of only about 1.3 each could potentially explain away the confidence interval (and a ratio of 1.5 removes the entire effect). Given the known associations between health-seeking behaviour, socioeconomic status, and both vaccination uptake and cardiovascular risk, an unmeasured confounder of this magnitude is fully plausible (especially when taking into account the large pre-matching differences we see in the sample characteristics). Therefore, reporting the E-value would make this vulnerability transparent and allow readers to judge for themselves whether matching on observables is sufficient to remove this threat (and I mean reporting these in the main text, with a small explanation for an uninitiated reader, not buried somewhere in the annex).

- Causal language

Abstract (p. 2)

"To mitigate these biases, we exploited a natural experiment created by the rapid transition from the live attenuated to the recombinant shingles vaccine"

"Exploited a natural experiment" implies a quasi-experimental identification strategy. As argued earlier, the design lacks a mechanism for as-if random assignment. More accurate would be: "We compared matched cohorts vaccinated before and after the transition".

Introduction (p. 3)

"By comparing cohorts who have both chosen to be vaccinated against shingles (with the live vaccine serving as a control given its lack of association with cardiovascular outcomes), this study design also eliminates the healthy-vaccinee bias."

"Eliminates the healthy-vaccinee bias" is a strong claim that is, in my view, incorrect. Both cohorts chose to be vaccinated, but the selection processes differed between eras (as argued above). "Reduces" or "attenuates" would be more defensible than "eliminates".
Additionally, the parenthesis that the live vaccine is a valid control because of its "lack of association with cardiovascular outcomes" relies entirely on one external study that uses a completely different sample and setting (Pomirchy et al 2025). This is a key inferential step that deserves more than a parenthetical citation. If Zostavax does have some cardiovascular effect (even a small one), then the interpretation of the entire study changes.

"Using a similar natural experiment, we have previously provided evidence that recombinant shingles vaccination may directly lower the risk of dementia."

The identical research design is used in Taquet et al. 2024. It is not a natural experiment there either. Also, note the contradicting tendencies in this sentence: on the one hand "natural experiment" indicates that their design can identify a causal effect, but then they qualify this with "may […] lower the risk", implying that the evidence perhaps is better understood

as correlational than causal. I'd suggest dropping "natural experiment" here and just saying that "Using a similar design, we have previously…".

Discussion (pp5-7)

"The reduction of risk is clinically meaningful: if confirmed in clinical trials, a 0.8% absolute difference in cumulative incidences of IHD and HF among adults over 60 years-old would translate into hundreds of thousands of cases prevented in the USA alone."

"Cases prevented" is explicitly causal language and is not motivated with the current research design. The entire sentence also needs to be further motivated. The study only estimates a Shingrix-vs-Zostavax difference. Zostavax is already being phased out globally. The relevant public health question going forward is therefore whether Shingrix protects against cardiovascular diseases compared to no shingles vaccination, which this study does not directly answer. The authors only indirectly assume that Zostavax == no shingles vaccine based on one external study conducted in a different setting. These underlying assumptions and the evidence that supports them should be spelled out more clearly when arguing for the practical public health relevance of the findings.

"our natural experiment mitigates these biases and provides more robust evidence for a cardiovascular protective effect of the recombinant shingles vaccine."

"Natural experiment" and "protective effect" both imply causality. Drop these. More motivated for the design is "Protective association".

"The stronger association for IHD and HF than for ischaemic stroke may indicate a cardiac-specific effect".

"Effect" implies causation. "Association" or "pattern" would be more appropriate.

"the recombinant vaccine induces immune-endothelial changes that are cardioprotective"

"Induces" and "are cardioprotective" both strongly imply causality. This sentence presents a specific biological mechanism as the explanation for an association that had not been shown to be causal. "The recombinant vaccine may induce immune-endothelial changes that could be cardioprotective" would at least signal uncertainty.

"Such immune changes might be time-limited, explaining the attenuation of the effect in the second half of the follow-up, and supporting studies investigating the effects of repeated vaccination at regular intervals."

"Explaining the attenuation of the effect" treats the attenuation as a feature of a causal mechanism rather than as a pattern in an observational association. "Consistent with the attenuation of the association" would be more accurate.

"our study is a natural experiment (in that treatment allocation is probabilistic and dependent on an external factor) and thus less prone to bias than conventional cohort studies, it does not imply that associations are causal"

If the term "natural experiment" is dropped, then this sentence appropriately highlights the limitations of the design. The problem is that it comes after extensive causal language in the preceding paragraphs, which undermines its effectiveness.

(Remarks on code availability)

Reviewer #2

(Remarks to the Author)
The authors have substantially addressed my prior concerns. The revised manuscript is improved, provides additional analyses addressing cohort comparability and residual confounding. It appropriately tones down causal claims.

One very minor suggestion - "eliminates the healthy-vaccinee bias" could be "substantially mitigates healthy-vaccinee bias" or similar

(Remarks on code availability)
Its a minimal piece of code that shows the approach for some of the main analyses. Minimal data, which is understandable given that patient level data cannot be disclosed. The code runs as it stands.

Reviewer #3

(Remarks to the Author)
The authors have responded to my earlier review. This is a valuable study and ready for publication.

(Remarks on code availability)

Open Access This Peer Review File is licensed under a Creative Commons Attribution 4.0 International License, which permits use, sharing, adaptation, distribution and reproduction in any medium or format, as long as you give appropriate credit to the original author(s) and the source, provide a link to the Creative Commons license, and indicate if changes were made.
In cases where reviewers are anonymous, credit should be given to 'Anonymous Referee' and the source.
The images or other third party material in this Peer Review File are included in the article's Creative Commons license, unless indicated otherwise in a credit line to the material. If material is not included in the article's Creative Commons license and your intended use is not permitted by statutory regulation or exceeds the permitted use, you will need to obtain permission directly from the copyright holder.
To view a copy of this license, visit https://creativecommons.org/licenses/by/4.0/

Recombinant shingles vaccination and lower risk of cardiovascular events

Response to reviewers

Fabiana Corsi-Zuelli, Fang Li, Rachel Upthegrove, John A. Todd, Betty Raman, Paul J. Harrison, Maxime Taquet

We were pleased to read the reviewers' positive and constructive comments on our paper. Below we provide a point-by-point response to every comment. In answering these comments, we have conducted seven additional robustness analyses, which we believe make the study significantly stronger. We also provide a more nuanced characterisation and interpretation of the strengths and limitations of the study design and its outcomes.

Reviewer #1

This study compared adults ≥60 vaccinated just before versus after the U.S. switch from live to recombinant shingles vaccine. The recombinant vaccine was associated with a 9% increase in cardiovascular diagnosis-free time over 7 years (RMTL ratio 0.91, 95% CI 0.88–0.95). It reduced risks of ischaemic heart disease and heart failure in both sexes, and ischaemic stroke in men, with additional reductions in atrial fibrillation but not myocarditis or peripheral arterial disease. Associations were strongest early and attenuated over time.

I believe that this contribution provides novel evidence on an important topic and is a contribution worth publishing. Clearly, if shingles vaccination truly has a substantial protective effect for cardio- and cerebrovascular disease, this would be important to know for physicians, patients, vaccination recommendation committees, and researchers studying the mechanisms that lead to such conditions. Having said this, I think it is absolutely critical that the manuscript undergoes major revisions (which I outline below), particularly with regards to the use of the term natural experiment, before it is considered for publication.

We thank the reviewer for recognising the importance of our study and for recommending to publish it. Below, we provide a point-by-point response to each of their comments. To answer them, we have conducted seven additional analyses that make the study more robust and help contextualise the results.

Major comments

1. The manuscript overstates the strength of the research design and uses overly strong causal language

1.1. The manuscript frames the study as a "natural experiment" in which vaccination timing "serves as a quasi-random assignment" and "eliminates healthy-vaccinee bias." It is my view that this characterization is not supported by the empirical evidence presented and should be changed.

We agree that accurate language is important. The term 'natural experiment' is used inconsistently in the literature. In the strictest sense of the term, a natural experiment is a situation in which there is random assignment of a treatment via a randomisation device, but this assignment is not under the control of the researcher (hence the qualifier 'natural')[1]. An example of such natural experiments is the assessment of the impact of change in wealth by observing lottery winners. However, the vast majority of 'natural experiments' (especially in medicine) do not enjoy this 'perfect randomisation' property. Instead, many rely on a treatment assignment that is under the control of an external factor which is the source of probabilistic assignment of treatments.

A formal definition of natural experiments has recently been provided and motivated by Titiunik[1]. It requires that treatment assignment:

1. is neither designed nor implemented by the researcher
2. is unknown to the researcher, and
3. is probabilistic by virtue of depending on an external factor.

Besides formalising the concept, this definition is helpful for two reasons. First, it draws a sharp distinction between natural experiments and randomised controlled experiments and keeps the latter as the gold standard for causal inference (which we agree with, as explicitly stated in our abstract: "These results justify clinical trials"). Second, it helps explain why natural experiments can be less biased than other 'traditional' observational studies (where the assignment mechanism is not known to depend on an external factor). The reason is that treatment assignment, by virtue of relying on an external factor, offers a plausible claim of being less confounded.

We believe our study meets these criteria and we have therefore kept the term 'natural experiment', not least because none of the additional 'diagnostic' analyses (suggested by the reviewer and conducted for this revised version) has revealed any residual confounding. However, natural experiments in this sense cannot be claimed to *demonstrate* causality which requires additional assumptions (notably, as highlighted by the reviewer below, that of conditional exchangeability). We have therefore made several key adjustments to the manuscript:

1. We have removed the term 'natural experiment' from the title to avoid giving the study design more prominence without contextualisation.

2. In the abstract, we have changed "To address these biases" by "To mitigate these biases" to reflect the fact that residual biases might still be present.

3. In the introduction, we have removed the statement "To start to provide causal evidence for the cardiovascular effect" and "Because there is no obvious reason for systematic differences between people who were vaccinated on either side of this time cut-off, vaccination timing serves as a quasi-random assignment that minimises confounding." Instead we have now written:

   Because there is no obvious reason for systematic differences between people who were vaccinated on either side of this time cut-off, this design mitigates biases that affect conventional cohort studies.

4. In the Discussion, we have replaced the sentence "By contrast, our natural experiment minimises these biases and starts to provide causal rather than correlational evidence for a cardiovascular protective effect of the recombinant shingles vaccine" by the following:

By contrast, our natural experiment mitigates these biases and provides more robust evidence for a cardiovascular protective effect of the recombinant shingles vaccine.

5. In the Discussion, we also explicitly highlight the need for additional assumptions to make causal claims, which we detail in a new Supplementary Note 3:

Third, while our study is a natural experiment (in that treatment allocation is probabilistic and dependent on an external factor[15]) and thus less prone to bias than conventional cohort studies, it does not imply that associations are causal. Causality requires additional assumptions (outlined in Supplementary Note 3 alongside supporting evidence and discussion of remaining uncertainties). In particular, while population drifts in measured characteristics (e.g. age) were adjusted for, changes in unmeasured characteristics cannot be excluded.

We believe these nuances significantly help contextualise the findings and the strengths and weaknesses of the study design. Overall, we believe our study represents an important contribution compared to traditional 'vaccinated-versus-control' designs that are considerably more prone to the sources of biases highlighted by the reviewer below.

On the healthy-vaccinee bias (defined as the systematic differences between vaccinated and unvaccinated people), we believe our study does remove it by comparing two cohorts who have both been vaccinated.

1.2. Supplementary Table 1 reveals substantial pre-matching differences between the April–September 2018 and April–September 2017 cohorts, most strikingly in age (69.27 vs. 65.48 years; SMD 0.63), alongside imbalances across a range of demographic and clinical characteristics. Far from being minor residual discrepancies, they indicate that the two groups were materially different before any adjustment. Matching can improve balance on observed covariates, but it does not transform a fundamentally non-exchangeable before/after comparison into a natural experiment.

Before matching, there was indeed a difference in age between cohorts. There were also differences in other clinical characteristics (e.g. skin cancer and ear/mastoid diseases both have SMD just over 0.1); however these are likely correlated with age as they disproportionately affect older people.

We attributed these differences to drifts in the population characteristics, i.e. secular trends in who gets vaccinated against shingles rather than abrupt, vaccine-type-specific shifts at the live/recombinant vaccine interface. This is based on data from CDC's National Health Interview Survey showing a gradual widening of the shingles vaccine coverage gap between older (>70, more likely to be vaccinated) and younger adults (60-69, less likely to be vaccinated). This increasing gap preceded the transition from the live to the recombinant shingles vaccine and there is no step change in age of uptake at the transition from the live to the recombinant shingles vaccine, i.e. from 2017 to 2018 (Fig. R1).

Redacted

Fig. R1 - Widening of the gap in shingles vaccine coverage between older and younger adults showing a gradual drift rather than a step change from 2017 to 2018 (source: NCHS, National Health Interview Survey, https://www.cdc.gov/nchs/products/databriefs/db370.htm)

We appreciate this reference was not included and is important for context and we have therefore added it in the Methods:

Evidence from the National Health Interview Survey[16] indicates a gradual change in the demographic characteristics of individuals receiving shingles vaccination over time, with increasing coverage among older (≥70 years) compared with younger (60–69 years) adults preceding the transition from live to recombinant vaccines. If unadjusted for, these drifts in the vaccinated population characteristics would affect our effect estimates when comparing people vaccinated in two different calendar periods.

These trajectories indicate that the inter-cohort differences observed in Supplementary Table 1 reflect broader secular changes in the demographic of those choosing to be vaccinated rather than abrupt selection at the vaccine interface.

In other words, this analysis supports the assumption that our analysis has conditional exchangeability (conditional on observed drifts in age and other characteristics). However, we agree that we cannot prove exchangeability and the absence of unmeasured confounders. We had already mentioned this in the Discussion and we have now made this more explicit in light of the pre-matching differences in characteristics:

[…] it does not imply that associations are causal. Causality requires additional assumptions (outlined in Supplementary Note 3 alongside supporting evidence and discussion of remaining uncertainties). In particular, while population drifts in measured characteristics (e.g. age) were adjusted for, changes in unmeasured characteristics cannot be excluded.

Importantly, all measured characteristics that were found to differ between cohorts before matching would tend to *increase* the risk of the cardiovascular outcomes among those vaccinated in 2018. If unmeasured confounders follow the same trend, they would tend to bias the estimate towards the null rather than away from it.

1.3. The quasi-random assignment assumption is further weakened by the specific context of the ZVL-to-RZV transition. This was not a neutral substitution: it followed updated recommendations from the Advisory Committee on Immunization Practices, which introduced preferential guidance for RZV and recommended vaccination of prior ZVL recipients.[1] At the same time, RZV uptake was plausibly selective. The vaccine requires a two-dose schedule, carries greater reactogenicity, and was introduced under conditions of limited supply with considerable public attention. All of this raises the threshold of effort and tolerance required for uptake and makes a random-assignment approximation implausible.

We agree that some of these factors influence which shingles vaccine people received. This would be most problematic if we had directly compared people who received ZVL to those who received RZV. However, our study compared *all* people who received their first shingles vaccination (either ZVL or RZV) between April and September 2018 to *all* people who received their first shingles vaccination between April and September 2017. By including all people in both groups, we limited the influence of such factors since people who elected to receive ZVL when RZV was already available (for reasons that might correlate with the outcome) would still be part of the '2018' cohort.

This limits but does not fully eliminate the influence of the selective RZV uptake. As such, our study substantially mitigates – but does not eliminate – bias compared to conventional vaccinated vs. control comparisons.

We have removed mention of 'quasi-random assignment' from the text, and we have added the helpful contextualisation from the reviewer in the limitation section of the Discussion:

In particular, while population drifts in measured characteristics (e.g. age) were adjusted for, changes in unmeasured characteristics cannot be excluded. ***Such unmeasured confounders are plausible given that the transition from the live to the recombinant vaccine followed updated recommendations from the Advisory Committee on Immunization Practices which might have influenced who got vaccinated.***

1.4. This concern is compounded by important data limitations. The EHR data contain no direct information on education, income, occupation, or living conditions. The only available proxy for socioeconomic disadvantage ("problems related to housing and economic circumstances") is extremely rare (<0.3%) and is therefore unlikely to capture meaningful variation in health-seeking behavior. Given well-established associations between socioeconomic position, healthcare access, and preventive behavior, residual confounding along these dimensions remains a central and unresolved concern.

Again, we agree with the reviewer. However, this would be more concerning for studies directly comparing vaccines rather than comparing timings of vaccination, highlighting the importance of our study design compared to the existing literature. Between April and September 2018, both vaccines remained available and if an individual chose/was offered the live vaccine during that time window (e.g. because of their socioeconomic background), they would still be included in the 2018 cohort.

In addition, while lifestyle factors are not explicitly captured in EHR, it is worth noting that they are not fully independent from clinical variables available in EHR data, such that clinical variables (e.g. obesity, diabetes, substance use disorder, etc) encode some information about lifestyle.

We had already included a sentence highlighting this limitation in the discussion:

"This study has several limitations beyond those inherent to electronic health record research (e.g. unvalidated diagnoses and ***limited socioeconomic and lifestyle data****; see Supplementary Note 1)."*

We have now also highlighted how this limitation can affect the assumption behind the natural experiment in Supplementary Note 3:

While the above evidence supports exchangeability on observed dimensions, the influence of several unmeasured factors cannot be fully ruled out. Socioeconomic status (education, income, occupation, neighbourhood deprivation) is not captured in EHR data, and lifestyle factors (smoking, diet, alcohol use) are only indirectly and imperfectly captured through related clinical variables (obesity, diabetes, substance use disorder, etc.). If they systematically differ between cohorts after matching, such unmeasured confounders may violate the exchangeability assumption and thus the causal interpretation of our findings.

1.5. The design is, in my view, most accurately described as a matched observational comparison across two vaccine-market periods. While matching improves balance on measured variables, it does not establish exchangeability, particularly in the presence of unmeasured socioeconomic and healthcare-access factors. The current causal language accordingly goes further than the evidence allows. Statements that the study provides "causal rather than correlational evidence" or that the design "eliminates healthy-vaccinee bias" are not supported by the identification strategy. The supplement appropriately acknowledges residual confounding, but this caveat is absent from the main text, where it is most needed. A more defensible framing would describe the findings as associations consistent with a protective effect, rather than causal estimates.

As mentioned in response to comment 1.1, we fully agree that more careful language was needed and we have made several changes in the title, abstract, introduction, methods, and discussion of the paper, as well as adding Supplementary Note 3. We have kept the term 'natural experiment' as we believe our study meets its definition[1] and helps frame the study in contrast to the existing literature. However, there is no more mention of causality in the manuscript, and the conclusion of the manuscript explicitly uses the term "associated".

1.6. It is also critical for the paper to report additional diagnostics on residual confounding. In particular, the authors should show if recent healthcare utilization, new diagnoses, or treatment changes showed different trends in the groups who received RZV and ZVL during the time before vaccination. This should be done by showing the time trajectories of preventive health indicators (influenza vaccination uptake, pneumococcal vaccination uptake, screening [e.g. colorectal cancer, skin cancer, breast cancer], new prescriptions of statins, anti-hypertensives or antidiabetic medications) and prior diagnoses of cardio-vascular diseases. Even if the two samples appear identical after matching on cumulative baseline indicators, this balance might mask different time trends prior to vaccination which would contribute to inter-group differences after vaccination. These diagnostics would not establish exchangeability but would offer more direct evidence on whether the matched groups were on comparable pre-vaccination health trajectories.

We thank the reviewer for suggesting additional diagnostic tests to assess differences in clinical trajectories pre-vaccination. We addressed it with two complementary additional analyses and neither of them reveals unmeasured confounding.

First analysis: different window for baseline covariates ascertainment

In our primary analysis, baseline covariates were defined as any prior occurrence before vaccination. This is because in TriNetX, a single time window can be defined for all covariates. To test whether cumulative-state matching masks differences in more recent trajectories, we re-ran the propensity-score matching and analysis using narrower time windows for baseline covariate ascertainment: 1, 2, 5, and 10 years before vaccination. We also included the number of visits as an additional covariate in these analyses to reflect healthcare use within the ascertainment windows.

All four time-window specifications yielded an RMTL ratio of 0.94 (all $P < 1 \times 10^{-5}$). Together, these analyses indicate that the primary finding is not an artifact of cumulative-state matching and is robust to recent-trajectory imbalances.

We have described this additional analysis in the Methods:

Analyses were repeated after […] (iii) using narrower ascertainment windows for baseline covariates (1, 2, 5, and 10 years before vaccination) while also adjusting for the number of health encounters during these time windows, to assess whether matching on cumulative-state covariates might mask differences in more recent pre-vaccination clinical trajectories

In the Results (in addition to being included in Table 1):

The association remained similar […] when different time windows were used to ascertain covariates (Table 1).

Second analysis: clinical trajectory in the year before vaccination

We further assessed whether the people vaccinated against shingles in the two calendar windows were on similar health trajectories before vaccination by comparing preventive health indicators and cardiovascular events (the primary outcome) in the year before vaccination. This was done after matching cohorts on covariates recorded up to 1 year before vaccination (rather than up to vaccination) to avoid conditioning on future events.

We examined all indicators suggested by the reviewer: influenza vaccination, pneumococcal vaccination, breast and bowel cancer screening, and prescriptions for statins, antihypertensives, and antidiabetic medications.

We first tested whether these indicators were affected by secular trends, which would limit their usefulness for comparing cohort trajectories. To do this, we compared their occurrence in the year before TDaP vaccination among individuals vaccinated in April-September 2018 versus April-September 2017. This is similar to the approach we already used in the manuscript for the assessment of secular trends in cardiovascular events. Many of the suggested indicators differed between these two calendar periods among TDaP vaccine recipients, indicating secular trends. This was expected, as some preventive interventions, such as cancer screening[2], tend to increase over time.

Two exceptions were influenza and pneumococcal vaccination, neither of which showed evidence of a secular trend, making them suitable indicators for testing systematic differences in preventive health behaviour between cohorts. In the year before shingles vaccination, the two cohorts in our primary analysis did not differ in their likelihood of receiving influenza vaccination (RMTL ratio 1.02, 95% CI 0.99–1.05; P=0.26) or pneumococcal vaccination (RMTL ratio 1.03, 95% CI 0.94–1.12; P=0.57).

To complement the indicators suggested by the reviewer, we also included 'preventive medicine services' (CPT codes 1013829 and 1013859) which encodes comprehensive preventive medicine evaluation and management of an individual (including history, examination, counseling/anticipatory guidance/risk factor reduction interventions, and the ordering of laboratory/diagnostic procedures) as it captures preventive health behaviour, is frequently coded (thus allowing sufficient statistical power to detect any difference) and has been used before in a study on shingles vaccination in TriNetX[3]. We found that it did not differ between cohorts in the year before shingles vaccination (RMTL ratio 1.02, 95% CI 0.96–1.08, P=0.58).

Finally, we found no difference in the primary cardiovascular composite endpoint in the year before vaccination (RMTL ratio 0.99, 95% CI 0.92–1.06, P=0.72).

These null associations with narrow confidence intervals strongly suggest that the two cohorts were following similar clinical trajectories before their shingles vaccination. This analysis is now described in the Methods:

We examined several indicators of preventive health use, including influenza and pneumococcal vaccination, prescriptions for statins, antihypertensives, and antidiabetics, and breast and bowel cancer screening. However, some of these may vary across calendar periods for reasons unrelated to shingles vaccination timing, limiting their value for this comparison. To identify indicators unaffected by such secular trends, we applied the same approach as above, comparing their frequency in the year before TDaP vaccination received in April-September 2018 versus April-September 2017. Only influenza and pneumococcal vaccination showed no systematic difference between the two periods, so we used these as indicators, together with a general code for preventive medicine services (CPT codes 1013829 and 1013859). Using the same approach, we also compared the risk of the primary cardiovascular endpoint in the year before shingles vaccination between the 2017 and 2018 cohorts to assess whether they were on similar cardiovascular health trajectories before vaccination.

In the Results:

In the year before vaccination, the two cohorts did not differ in preventive healthcare use and in the risk of cardiovascular events (Supplementary Table 6).

And Discussion:

However, the absence of association with a negative control outcome, the lack of difference in clinical trajectories in the year before vaccination, the similarities in preventive healthcare use during follow-up, and the similar risk of non-cardiovascular death provide support for an absence of overt unmeasured confounders.

1.7. Finally, while similar "natural experiment" language has been used in the authors' prior work employing a comparable design,[2] this previous language was, in my opinion, inappropriate, and the precedent does not justify the same language in the current study without substantial additional evidence corroborating the underlying assumptions. Specifically, previous studies have demonstrated that vaccination timing is associated with education, income, uptake of other vaccinations (e.g., influenza),[3] in addition to ethnicity and self-rated health.[4]

We hope the answers to previous points provide some clarification on the use of the term natural experiment. We also believe we now provide more corroborating evidence supporting underlying assumptions, summarised in Supplementary Note 3 alongside remaining uncertainties.

We note that, in reference 3, the gap between early and late adopters was approximately 5 years, far greater than the 1-year difference between our two cohorts. One of the main absolute differences reported in that study was influenza vaccine uptake. In contrast, our cohorts showed little difference in prior influenza vaccination, even before matching and, as noted above, influenza vaccination rates in the year immediately preceding shingles vaccination were also very similar between cohorts.

Reference 4 focuses on differences between vaccinated and unvaccinated individuals, rather than on vaccination timing. It is therefore not directly applicable to our study and further underscores the importance of study designs that go beyond simple vaccinated-versus-unvaccinated comparisons.

2. Outcome definitions require clearer justification

2.1. The composite endpoint of ischemic heart disease, ischemic stroke, and heart failure is broadly motivated but requires more explicit justification at several points.

The omission of transient ischemic attack (TIA) is difficult to reconcile with the existing literature, which consistently treats stroke and TIA as related outcomes and reports elevated risk following herpes zoster.[5-7] At a minimum, TIA should be included as a secondary outcome or its exclusion explicitly justified.

Many thanks for this suggestion. The reason for excluding TIA from our primary outcome is that misdiagnosis of TIA is more common than that of strokes due to higher diagnostic challenges compared to stroke[4].

However, we have now added it as a secondary outcome. The RMTL ratio comparing those vaccinated in April–September 2018 with those vaccinated in April–September 2017 was 0.95 (95% CI 0.86–1.06; P=0.35), similar to the estimate for ischaemic stroke. As with ischaemic stroke, the event rate was

relatively low (2.63% in the recombinant cohort vs 3.17% in the live-attenuated cohort), which may have limited power to detect differences between groups. We have updated the Methods accordingly and added the corresponding results to Table 1 and Extended Data Figure 3.

2.2. The inclusion of heart failure is less clearly supported. The association between herpes zoster and heart failure is weaker and partly bidirectional, with evidence pointing both to increased HF risk following zoster and to increased zoster risk among individuals with pre-existing HF.[8,9] The manuscript should clarify whether heart failure here refers to incident disease, worsening of pre-existing disease, or HF arising as a complication of another cardiovascular event captured within the same follow-up window.

Our rationale for the selection of the components of the primary outcome was to include cardiovascular events that have been found to be possibly affected by the recombinant shingles vaccine according to prior observational studies (references 2–4 in our manuscript). The composite of ischaemic heart disease, heart failure, and stroke is also often used as a primary outcome in prospective clinical trials (e.g. the landmark SPRINT trial[5]).

We have now clarified this in the methods:

These were selected as they were found to be associated with the recombinant shingles vaccine in prior observation studies[2–4] and are often combined in clinical trials of cardiovascular interventions[17].

The primary outcome was incidence of cardiovascular events in people who had not had a recorded diagnosis of heart failure, ischaemic stroke, or ischaemic heart disease before vaccination. People could have developed one cardiovascular event (e.g. IHD) and then another (e.g. HF) and the first would count in the survival analysis of the composite endpoint. However, in the analysis of each component of the composite endpoint, people could have had another cardiovascular event during follow-up, and it is not possible to ascertain whether one is a complication of the other. We have now clarified this in the Methods:

We also report each component of the composite primary endpoint separately. While one component (e.g. heart failure) could be a complication of another (e.g. ischaemic heart disease) occurring in the same follow-up, this could not be ascertained in the data.

2.3. Finally, the exclusion of hemorrhagic stroke warrants explicit justification. Meta-analytic evidence identifies a significant association between herpes zoster and hemorrhagic stroke,[10] meaning this outcome is not negligible a priori. In my view, hemorrhagic stroke should be included at least in secondary analyses.

We thank the reviewer for this suggestion. As outlined above, this was not part of our rationale for inclusion in the primary outcome. However, we have now included it as a secondary outcome. There was no difference between cohorts (RMTL ratio 1.08, 95% CI 0.93–1.25; P = 0.33). The event rate was relatively low (1.55% in those vaccinated in April-September 2018 and 1.80% in those vaccinated in April-September 2017). This outcome definition has been added to the Methods, and the corresponding results are now reported in Table 1.

3. The primary time-to-event analysis should account for competing risks. The analysis uses Kaplan–Meier methods in a cohort where death is a competing event of considerable magnitude. Because individuals who die cannot subsequently experience cardiovascular events, standard survival methods are misaligned with the clinical estimand of interest. In my view, a competing-risks framework (e.g., Aalen–Johansen estimators combined with cause-specific or Fine–Gray models) should be used in the primary analysis. The composite of the primary endpoint and death does not resolve this issue; it changes the estimand rather than addressing the underlying problem. Given the length of follow-up and the incomplete mortality capture inherent in TriNetX, this issue is particularly consequential.

We thank the reviewer for this suggestion. We have now performed a competing-risks analysis on the same cohort used in the primary analysis, comprising three complementary steps.

1. Aalen–Johansen cumulative incidence function

We first estimated the cumulative incidence function (CIF) of the composite cardiovascular endpoint using the Aalen–Johansen (AJ) estimator, which treats non-cardiovascular death (i.e. death occurring before any recorded cardiovascular event) as a competing risk rather than as censoring. Gray's non-parametric test for equality of the two CIFs rejected the null hypothesis ($\chi^2 = 10.61$, df = 1, $P = 1.12 \times 10^{-3}$). For non-cardiovascular death, Gray's test did not detect a difference ($\chi^2 = 0.81$, $P = 0.37$). This suggests that the cohorts are balanced with respect to non-cardiovascular mortality and argues against differential mortality being an explanation for the difference in cardiovascular outcomes.

2. Fine–Gray sub-distribution hazard model and time-segmented analysis

We next fitted a Fine–Gray sub-distribution hazard model. The global sub-distribution hazard ratio (sHR) for the composite cardiovascular endpoint was 0.94 (95% CI 0.91–0.97, P < 0.00001). However, scaled Schoenfeld residuals indicated violation of the proportional-hazards assumption so the global sHR is of limited interpretative value. We therefore extended the Fine–Gray model to a time-segmented specification across four follow-up windows and found that, consistent with the primary finding, there is a significant association that subsides during the second part of the follow-up (see Supplementary Table 3 copied below for convenience).

Supplementary Table 3. Time-segmented Fine–Gray sub-distribution hazard ratios for the composite cardiovascular endpoint accounting for death as a competing risk.

Interval	Fine-Gray sHR(95%CI)	P value
0–1 year	0.85 (0.78–0.94)	0.0050
1–3 years	0.82 (0.77–0.88)	7.51×10^{-9}
3–5 years	1.01 (0.95–1.08)	0.76
5–7 years	1.04 (0.98–1.10)	0.22

3. Aalen–Johansen-based RMTL

Finally, to align the competing-risks framework with the restricted-mean-time-lost metric used in our primary analysis, we computed the Aalen–Johansen-based RMTL (AJ-RMTL) ratio. The AJ-RMTL ratio at seven years was 0.90 (95% CI 0.87–0.93, P = 8.4×10^{-9}) for the composite endpoint (which is very similar to our primary analysis) and 1.00 (95% CI 0.91–1.14, P = 0.86) for non-cardiovascular death.

Taken together, the Aalen–Johansen CIF with Gray's test, the time-segmented Fine–Gray model, and the AJ-RMTL analysis yield conclusions similar to our primary findings: the recombinant shingles vaccine is associated with a lower risk of cardiovascular events over seven years; the association is concentrated in the first part of the follow-up; and there is no evidence of differential non-cardiovascular mortality between cohorts.

We elected to keep this competing-risks analysis as a secondary analysis due to its higher complexity and to keep the paper within the word count limit of a Brief Communication. We have added the following to the Methods section of the revised manuscript:

To account for competing risks by non-cardiovascular death, we also estimated the cumulative incidence function (CIF) of the composite cardiovascular endpoint using the Aalen–Johansen estimator. Equality of CIFs between cohorts was tested with Gray's non-parametric test. Sub-distribution hazard ratios were obtained from a Fine–Gray model[18] fitted via the weighted Cox reformulation of Geskus with cluster-robust variance; the proportional-hazards assumption was assessed using scaled Schoenfeld residuals, and when violated, we fitted a time-segmented Fine–Gray model across four follow-up windows (0–1, 1–3, 3–5, 5–7 years). For consistency with the primary analysis, we also report the Aalen–Johansen-based restricted mean time lost (AJ-RMTL) ratio by direct integration of the Aalen–Johansen CIF curves.

The corresponding findings are presented in Table 1 and Supplementary Table 3 with the following accompanying text in the Results:

The association remained similar when accounting for death as a competing risk (and there was no association with non-cardiovascular death; Table 1 and Supplementary Table 3).

4. Cohort attrition and healthcare engagement require explicit assessment.

4.1. The manuscript does not report retention or loss to follow-up, despite prior work using the same data source documenting substantial attrition.[2] In EHR-based analyses, continued observation depends on ongoing interaction with healthcare systems and is therefore likely informative rather than ignorable.

I believe that the authors should report retention over time, compare follow-up distributions between groups, and assess robustness to differential attrition. Without such diagnostics, long-term comparisons may reflect selection into continued observation rather than causal effects, a concern that appears particularly relevant given the attenuation of estimated effects beyond approximately 3.5 years.

This was indeed missing from our original submission. We now report, for each year of follow-up the number of events, the number of people lost to follow-up, and total person-years of observation. This is added as Supplementary Table 4, copied below for convenience.

As can be seen, the available person-years of follow-up were very similar between the two cohorts every year with a small increase in censoring rate during the last year of follow-up among those vaccinated in 2018. This is expected since the last year of follow-up for that cohort coincides with when the study was conducted, so that individuals who had not made a recent healthcare encounter were censored.

This is also reflected in the distributions of follow-up (Fig. R2 below) showing that many had all 7 years of follow-up (note the log-scale for the y-axis) with slightly more people being censored sooner among those vaccinated in 2018 than among those vaccinated in 2017.

Supplementary Table 5. Follow-up time in person-years, number of composite cardiovascular endpoint cases, and number lost to follow-up (e.g., because they made no further contact with their healthcare provider or moved to a different area). These data are presented for each year of follow-up and for both the primary analysis and after alignment of the follow-ups.

**Primary analysis**	**Year 1**	**Year 2**	**Year 3**	**Year 4**	**Year 5**	**Year 6**	**Year 7**
**Vaccinated in Apr-Sep 2017**							
Person-years	31,206	29,625	28,006	26,392	24,707	22,691	20,448
Events	1,015	1,012	923	920	1,056	1,177	1,134
Censorings	549	562	787	644	807	953	1,173
**Vaccinated in Apr-Sep 2018**							
Person-years	31,288	29,705	28,185	26,519	24,509	22,235	19,180
Events	912	809	763	1,000	1,026	1,135	1,098
Censorings	609	852	667	908	1,048	1,391	3,098
**Aligned follow-up**	**Year 1**	**Year 2**	**Year 3**	**Year 4**	**Year 5**	**Year 6**	**Year 7**
**Vaccinated in Apr-Sep 2017**							
Person-years	30,455	27,444	24,612	21,829	18,875	15,753	12,242
Events	987	937	797	764	802	813	694
Censorings	2,028	2,063	1,945	2,114	2,204	2,424	3,422
**Vaccinated in Apr-Sep 2018**							
Person-years	30,455	27,444	24,612	21,829	18,875	15,753	12,242
Events	888	744	669	846	794	807	724
Censorings	2,127	2,256	2,073	2,032	2,212	2,430	3,392

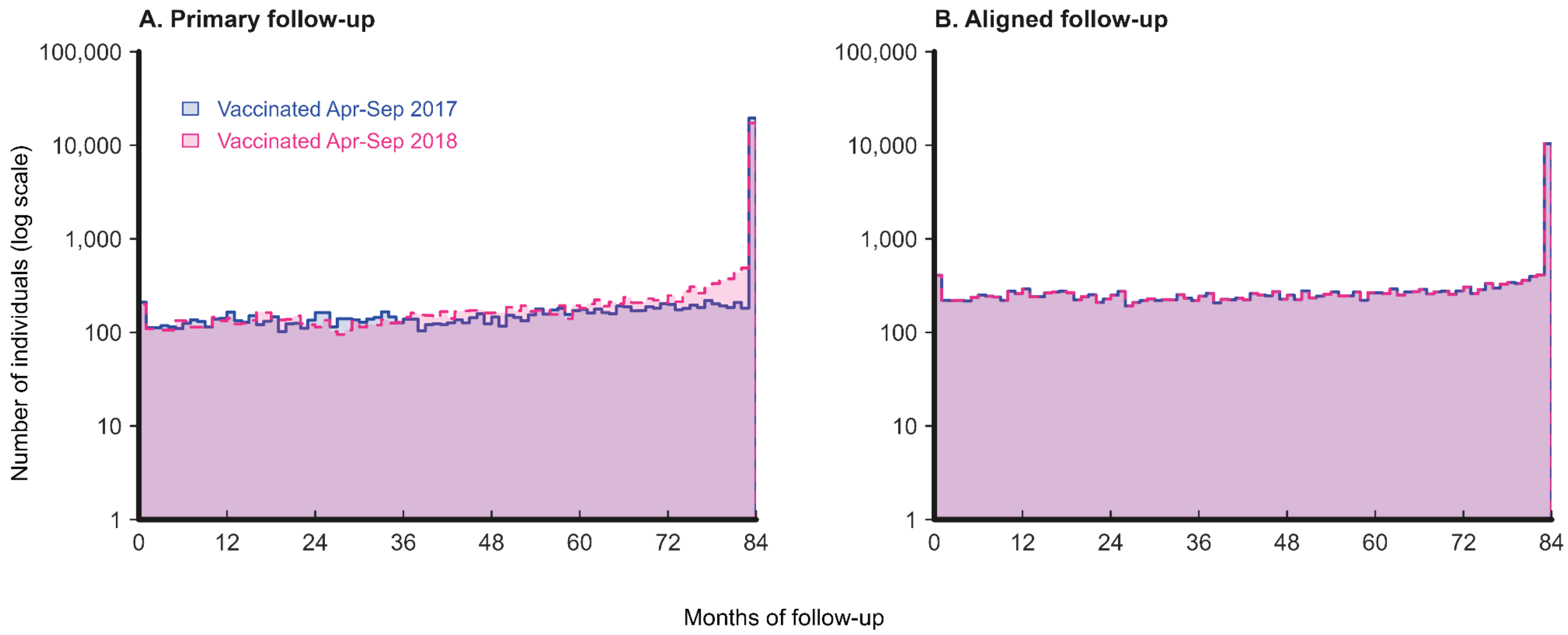

Fig. R2. Distribution of follow-up time per individual in the primary and aligned-follow-up analyses.

To assess whether informative censoring affected the results, we performed a robustness analysis in which follow-up was aligned between the two cohorts. For each pair of individuals (one per cohort), the longer follow-up time was censored to the shorter one. Because TriNetX does not release pair-level matching information for privacy reasons, this alignment was done at the cohort level by randomly pairing individuals, ensuring identical follow-up distributions in the matched cohorts. We used the same approach in our previous study of shingles vaccination and dementia[6].

Within these cohorts with aligned follow-up, the RMTL ratio for the primary outcome was found to be very similar to that in the primary analysis: 0.92 (95% CI 0.89–0.96, $P=1.9 \times 10^{-5}$), providing further evidence that informative censoring does not account for our findings.

Our understanding is that if bias by selection into continued observation was driving the protective association, the effect would be absent or weaker at early follow-up, when retention is highest and selection is minimal, and to grow as attrition accumulates whereas we observed the opposite. We found that the same attenuation of the association was observed when the follow-ups were aligned between cohorts: RMTL ratio 0.86 (95% CI 0.81−0.90) for the first half of follow-up and 1.03 (95% CI 0.97−1.09) for the second half.

We have added the following to the Methods section of the revised manuscript:

Analyses were repeated after […] (iv) aligning follow-up times at the cohort level (by trimming follow-up to the shorter member's observed follow-up in randomly selected pairs of individuals) as in our previous study[5] to assess robustness to cohort attrition and informative censoring.

And in the Results:

The association remained similar […] when the amount of follow-up time was matched between cohorts (Supplementary Table 5).

4.2. This section also provides an opportunity to examine potential post-vaccination differences in health-seeking behavior. If RZV recipients differ systematically in healthcare engagement, this may be reflected in their subsequent utilization of other vaccines (e.g., pneumococcal or TDaP) and preventive services. Examining such patterns is, in my view, important and would provide indirect evidence on residual healthy-user bias as well as help contextualize the observed outcome trajectories.

We agree that this is a valuable additional analysis to 'diagnose' the presence of residual confounding. We considered the same preventive health indicators as in our response to comment 1.6 above, except that this time we assessed them during the follow-up rather than in the year before vaccination.

As for our answer to comment 1.6, we first assessed whether there was any sign of secular trends in these outcomes by comparing their occurrence in the 7 years following TDaP vaccination received in April-September 2018 vs. April-September 2017. In doing so, we found that the following could be used as reliable markers of healthcare engagement (not confounded by secular trends): bowel cancer screening and anti-hypertensive prescription. As in our answer to comment 1.6, we also included preventive medicine services as an additional indicator of preventive health behaviour.

We found no difference between cohorts in bowel cancer screening (RMTL ratio 1.00, 95% CI 0.97–1.02), anti-hypertensive prescription (RMTL ratio 1.00, 95% CI 0.99–1.01), and preventive medicine services (RMTL ratio 1.01, 95% CI 0.98–1.04). This provides further support for the lack of obvious unmeasured confounding effect due to differential preventive service use.

We have now described this analysis in the Methods:

Similarly, we assessed preventive healthcare use between cohorts during the follow-up. We considered the same preventive healthcare indicators as above and selected those that showed no secular trends (as detected by comparing cohorts who received TDaP vaccines). This led to the selection of bowel cancer screening (ICD-10 CM Z12.11) and antihypertensive prescription (VA CV100 or CV200 or CV800 or CV701 or CV805), together with a general code for preventive medicine services (CPT codes 1013829 and 1013859).

And report the findings in the Results:

Likewise, there was no significant difference between cohorts during follow-up in terms of preventive health indicators (Supplementary Table 7).

5. Potential confounding from the COVID-19 pandemic. Outcome trajectories show a structural change at approximately 500 days of follow-up, corresponding to the onset of the COVID-19 pandemic. Pandemic-related declines in cardiovascular hospitalizations have been well documented[11,12] and likely reflect reduced care-seeking and altered diagnostic intensity rather than true changes in event incidence. The different patterns observed across outcomes (e.g., more abrupt for ischemic heart disease and heart failure, more gradual for stroke) introduce calendar-time confounding, as the two cohorts encounter the pandemic at different stages of follow-up. So, I would strongly recommend sensitivity analyses that restrict follow-up to before March 2020.

We thank the reviewer for this suggestion. We have now added a robustness analysis in which the follow-up is restricted to before the beginning of the pandemic. We found that associations were similar as in the primary analysis for both the composite endpoint and its individual components.

We have introduced this analysis in the Methods:

Analyses were repeated after […] (ii) limiting follow-up to the first 518 days so that it occurred entirely before the COVID-19 pandemic

The association for the primary endpoint is reported in Table 1 with the individual components reported in Supplementary Table 4 (copied below for convenience). We also refer to these associations in the Results section:

The association remained similar […] when the follow-up period was entirely before the COVID-19 pandemic (Supplementary Table 4)[…] (Table 1)

Supplementary Table 4. Sensitivity analysis restricting follow-up to before March 2020

Outcome	RMTL ratio (95% CI)	P value
Composite endpoint	0.89 (0.82–0.97)	0.0068
Ischaemic heart disease	0.88 (0.80–0.97)	0.0078
Heart failure	0.86 (0.75–0.99)	0.038
Ischaemic stroke	0.91 (0.75–1.11)	0.37

6. Estimand and external validity should be clarified. The matched analysis identifies an effect for the overlap population rather than for the full population of vaccine recipients. Given the substantial pre-matching differences documented in Supplementary Table 1, this distinction is consequential and should be stated explicitly. The manuscript should, in my opinion, define the estimand clearly and avoid generalizing findings to broader populations without appropriate qualification.

The reviewer is correct. We have now clarified the estimand in the Methods as follows:

Because after 1:1 matching, both cohorts are restricted to individuals who had a match in the other cohort, the estimand is best interpreted as the average treatment effect in the overlap population, in an intention-to-treat approach where treatment allocation depends on vaccination timing.

7. Effects on other outcomes should be documented. The fact that the authors observe an effect on cardiovascular outcomes in addition to the prevention of dementia raises questions about whether patients who received RZV are less likely than ZVL-receivers to be diagnosed with every kind of clinical condition in the follow-up period. Barring the possibility that RZV is a miracle cure for all diseases, such a set of results would imply there exist unobserved differences between patients who received RZV and ZVL not accounted for in the propensity-score matching algorithm.

To assuage these concerns, I believe it is critical that the authors show whether there are differences between RZV and ZVL for each condition in the Charlson Comorbidity Index or the, say, 20 most common diagnoses in their data.[13] It would be very informative to show if the effect of HZ-vaccination on cardio-vascular conditions was specific or if similar associations appear with respect to other common diagnoses.

We appreciate the reviewer's comment. However, finding appropriate negative control outcomes when the exposure is vaccination and the primary outcome is cardiovascular disease is challenging for four reasons:

1. Some outcomes may be directly affected by cardiovascular events and their treatments. For instance, following a cardiovascular event, many will receive long-term aspirin which is a major risk factor for peptic ulcers[7] (one of the components of the Charlson Comorbidity Index [CCI]). By the same token, the risk of renal diseases (another component of the CCI) can also be increased following cardiovascular events since aspirin (and other drugs used in this context) can directly injure the kidneys.

2. Some conditions may also be direct consequences of cardiovascular disease. For example, the recommended coding for chronic pulmonary disease in the CCI includes pulmonary heart disease[8]. Although excluding subcodes with cardiac aetiology might seem like a possible workaround, misdiagnosis and miscoding within a diagnostic category mean that we cannot be certain to have excluded all events with cardiovascular causes.

3. There is growing evidence that vaccination against one pathogen may protect against another unrelated virus[9]. Therefore any outcome that is caused or triggered by a viral infection may not serve as a negative control outcome.

4. Even for conditions that do not directly result from cardiovascular events, their apparent risk assessed in electronic health records might be raised following a cardiovascular event due to increased detection of that condition. For instance, most patients would have HbA1c (screening for diabetes) and liver function tests measured as part of the diagnostic workup or in the aftermath of a cardiovascular event, which could result in artefactually lower risk of such outcomes following any intervention that prevents cardiovascular outcome.

Most components of the CCI are therefore unsuitable as negative control outcomes. The only possible exception was solid tumours, which did not differ between groups (RMTL ratio 1.02, 95% CI 0.98–1.07; P=0.38). Expanding the outcome to all neoplasms, including benign lesions such as melanocytic naevi that are often detected incidentally and may reflect health-seeking behaviour, also yielded a null result (RMTL ratio 0.98, 95% CI 0.96–1.01; P=0.14). We have not included these additional analyses in the current manuscript because they require more nuanced justification than the Brief Communication format allows, but we would be happy to add them if the editor or reviewer feels this would be helpful.

Because of the difficulties to identify relevant negative control outcomes, we had selected a pre-specified negative control outcome made of a composite of acutely painful events that cannot be traced back to cardiovascular diseases and its treatments. We reported no significant association for this outcome.

Of note, we believe that any cardiovascular protection conferred by the recombinant shingles vaccine could plausibly contribute to the observed protection against dementia, given that many dementia cases reflect mixed Alzheimer and vascular pathology. This was part of our motivation for examining

cardiovascular outcomes. We therefore do not view these associations as independent signals of a broader, non-specific health benefit, but rather as potentially related effects that may lie along the same causal pathway, with reduced cardiovascular risk contributing to lower dementia risk.

8. The manuscript should more clearly delineate what matching can and cannot achieve
Throughout the manuscript, propensity-score matching appears to be invoked as a remedy for concerns broader than covariate balance.[14] Matching improves comparability on observed characteristics but does not address unmeasured confounding, differences in diagnostic intensity, or compositional changes in the vaccinated population across calendar periods. The manuscript would, in my view, benefit from a cleaner separation between the contribution of covariate adjustment and the requirements for causal identification, and should avoid implying that the former is sufficient for the latter.

We agree with the reviewer. As noted above, we have now added a section in the Supplement (Supplementary Note 3) where we clearly outline the assumptions behind a causal interpretation of our findings and the limitation of propensity-score matching in that regard. Specifically, we wrote:

While the above evidence supports exchangeability on observed dimensions, the influence of several unmeasured factors cannot be ruled out. Propensity-score matching can only achieve balance of measured covariates and the analysis of secular trends comparing TDaP vaccine recipients does not establish exchangeability between shingles vaccine cohorts. Socioeconomic status (education, income, occupation, neighbourhood deprivation) is not captured in EHR data, and lifestyle factors (smoking, diet, alcohol use) are only indirectly and imperfectly captured through related clinical variables (obesity, diabetes, substance use disorder, etc.). If they systematically differ between cohorts after matching, such unmeasured confounders may violate the exchangeability assumption and thus the causal interpretation of our findings

In addition, we have explicitly stated in the Methods within the main manuscript:

Adjustment for these covariates seeks to achieve conditional exchangeability of the cohorts. Importantly, it cannot achieve exchangeability if there are unmeasured confounders (Supplementary Note 3).

Minor comments
The Discussion notes early divergence in outcome trajectories but moves quickly towards a biological interpretation. It would be important to acknowledge upfront that early divergence is also consistent with residual confounding or systematic differences in healthcare utilization between the two cohorts.

Early divergence of Kaplan-Meier curves can indeed be a sign of unmeasured confounding. However, it is also worth noting that residual confounding by time-invariant confounders (e.g. genetic variants or stable socioeconomic factors) tend to have ongoing influence on the outcome and so contribute to ongoing divergence of the curves (unlike the convergence in the second half of follow-up seen here).

We have now acknowledged this in the Discussion (added in the limitation section where we think it fits better than in the paragraph on possible mechanisms):

Early divergence of Kaplan-Meier curves, while biologically plausible, can be a sign of unmeasured confounding (although later convergence of these curves is incompatible with ongoing influence of time-invariant confounders).

The TDaP comparison is a useful diagnostic tool for ruling out gross secular trends, but it does not establish exchangeability between the shingles vaccine cohorts. This distinction should be made more explicit.

We agree and this is now acknowledged in Supplementary Note 3 where we discuss each assumption behind the natural experiment. Specifically, we write:

Propensity-score matching can only achieve balance of measured covariates and the analysis of secular trends comparing TDaP vaccine recipients does not establish exchangeability between shingles vaccine cohorts.

There is meaningful heterogeneity in temporal trajectories across outcomes, particularly for ischemic heart disease, which shows an early decline followed by a reversal not observed in other outcomes. This pattern is not clinically intuitive and warrants more careful discussion. It may reflect changes in the composition of the risk set over time, or the well-documented sensitivity of IHD coding to healthcare utilization.

We appreciate that the curves in Fig. 1C might make it look like there is a reversal for ischaemic heart disease. However, the 95% CI around the curve (not included in the original submission, for clarity, but depicted in Fig. R3 below) is broad and compatible with a plateauing of the difference in cumulative incidence (which we already discussed in the Discussion). This is also reflected in the hazard ratio for the second half of the follow-up which is not significantly different from 1. We would therefore not want to overly speculate that there is any reversal of the protective association.

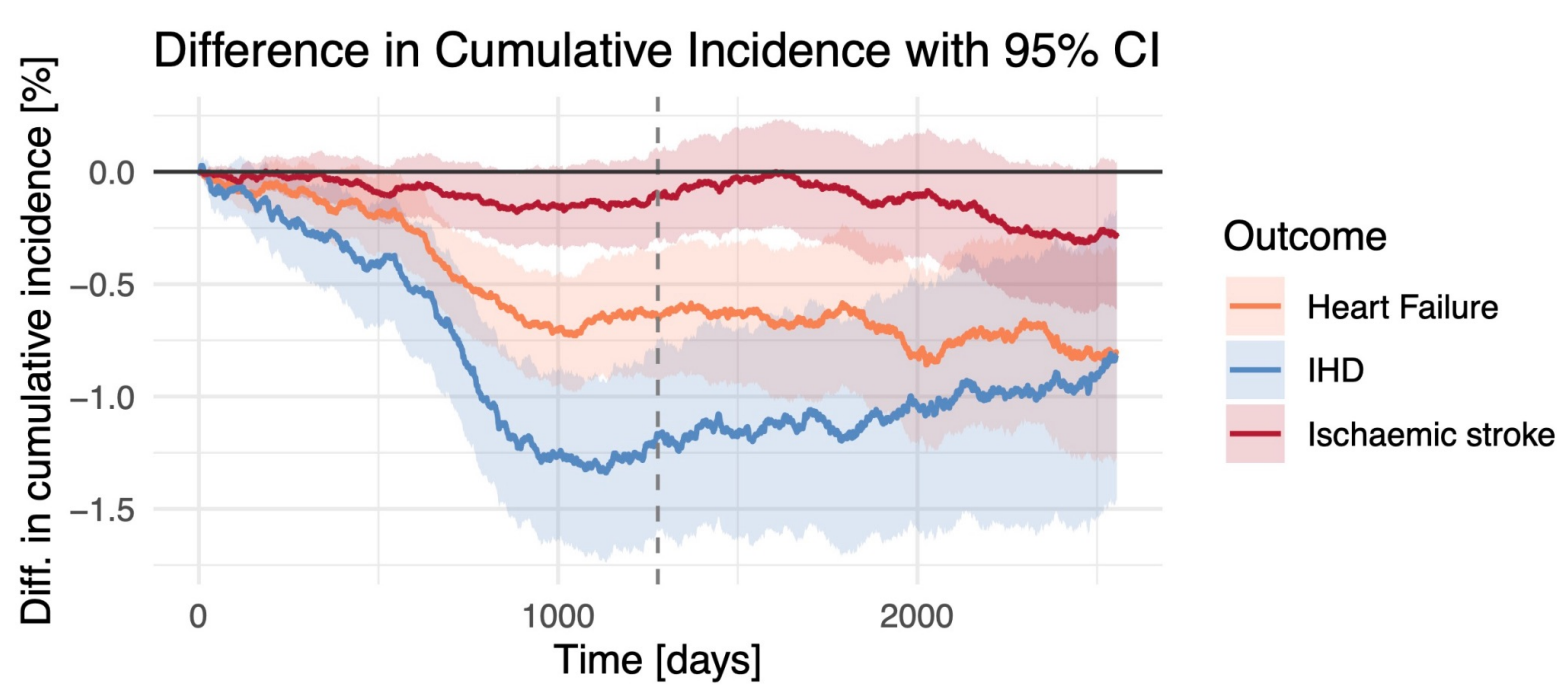

Fig. R3 - Same figure as Fig. 1C in the manuscript but with 95% CI overlaid.

The manuscript would benefit from a clearer account of follow-up and censoring assumptions, particularly given that continued observation depends on ongoing interaction with the participating healthcare organizations.

This is now addressed as a response to question 4.1 above wherein we show that the two cohorts have similar distributions of follow-up times and that results remain unchanged when follow-up times are aligned between cohorts.

Finally, there appears to be a coding discrepancy in the supplement, where both acute pancreatitis and acute cholecystitis are assigned ICD-10 code K81.0.

We thank the reviewer for spotting this typo which has now been corrected (acute pancreatitis has ICD-10 code K85).

Reviewer #2

Summary
This study uses the switch from live to recombinant zoster vaccination in the US as a natural experiment to compare cardiovascular outcomes. In matched adults aged 60 and over, the recombinant vaccine was associated with a lower risk of the composite cardiovascular endpoint over 7 years. The association was clearest for IHD and HF, weaker for stroke, and attenuated over time. Secondary and control analyses were broadly consistent with the main findings.

Originality/significance
The design is more robust than prior vaccinated versus unvaccinated comparisons and addresses healthy vaccinee bias. However, as mentioned below, I am not sure what the quasi-randomised design is testing, since this is now a comparison of two vaccines rather than vaccination versus no vaccination. Is the question whether any zoster vaccine might prevent CVD, or whether recombinant vaccine is more protective than live vaccine? If it is the latter, the main justification given in the introduction feels somewhat tangential.

We thank the reviewer for recognising that the approach is robust and addresses the important healthy vaccinee bias.

The question that this study answers is indeed whether the recombinant shingles vaccine is associated with lower risk of cardiovascular events than the live shingles vaccine. However, as there is already strong evidence from a natural experiment that the live shingles vaccine provides no protection against cardiovascular events[10], the live vaccine can be considered a control group in whom the risk of cardiovascular events is similar to that in the general population. We have now clarified this in the introduction:

*By comparing cohorts who have both chosen to be vaccinated against shingles (**with the live vaccine serving as a control given its lack of association with cardiovascular outcomes[5]**), this study design also eliminates the healthy-vaccinee bias.*

Data/methods
Large EHR dataset and extensive matching are strengths. The natural experiment is plausible but the assumptions is that people vaccinated just before and just after the transition should be comparable. Residual confounding, selection around the transition, and incomplete capture of care remain possible. I really appreciate the use of a negative control outcome (acutely painful conditions) to balance this.

We agree with the reviewer that residual confounding is a possibility. We have now improved the study in two ways in that regard: by providing additional tests of similarity of cohorts around the transition, and by modifying the text to better reflect the risk of unmeasured confounding.

Additional analyses

To provide further support against such residual confounding, we have conducted an extensive set of additional analyses (in addition to the existing analysis of the negative control outcome and the exclusion of secular trends). None of them revealed unmeasured confounding. Specifically, we have shown the

following:

1. In the year prior to vaccination, the risk of cardiovascular outcomes was similar between cohorts (RMTL ratio 0.99, 95% CI 0.92–1.06, P=0.72).

2. In the year prior to vaccination, the use of preventive health interventions as assessed by several preventive health indicators was similar between cohorts. These health indicators were selected to show no secular trend between calendar periods so that any difference could be attributed to selection around the transition. Details of this analysis are provided in response to Reviewer 1's Comment 1.6.

3. During follow-up, no difference between cohorts was observed in their use of preventive health interventions. Details of this analysis are provided in response to Reviewer 1's Comment 4.2.

4. In a formal competing risk analysis (using Aalen–Johansen estimators combined with cause-specific or Fine–Gray models), the risk of non-cardiovascular death was found to be very similar between cohorts (RMTL ratio 1.00, 95% CI 0.91–1.14, P=0.86).

5. In an additional set of robustness analyses, we limited the time window for ascertainment of baseline covariates and added the number of visits within each time window as an additional covariate and found that the associations remained unchanged.

6. The impact of the COVID-19 pandemic hitting the cohorts at different points of their follow-up was found not to affect the results, as shown by limiting the follow-up to be entirely before the pandemic.

7. The impact of potential differences in follow-up times was also assessed and found to have no impact on the main results.

These analyses are described in the Methods:

Analyses were repeated after (i) stratification by sex; (ii) limiting follow-up to the first 518 days so that it occurred entirely before the COVID-19 pandemic; (iii) using narrower ascertainment windows for baseline covariates (1, 2, 5, and 10 years before vaccination) while also adjusting for the number of health encounters during these time windows, to assess whether matching on cumulative-state covariates might mask differences in more recent pre-vaccination clinical trajectories; and (iv) aligning follow-up times at the cohort level (by trimming follow-up to the shorter member's observed follow-up in randomly selected pairs of individuals) as in our previous study[6] to assess robustness to cohort attrition and informative censoring.

To account for competing risks by non-cardiovascular death, we also estimated the cumulative incidence function (CIF) of the composite cardiovascular endpoint using the Aalen–Johansen estimator. Equality of CIFs between cohorts was tested with Gray's non-parametric test. Sub-distribution hazard ratios were obtained from a Fine–Gray model[18] fitted via the weighted Cox reformulation of Geskus with cluster-robust variance; the proportional-hazards assumption was assessed using scaled Schoenfeld residuals, and when violated, we fitted a time-segmented Fine–Gray model across four follow-up windows (0–1, 1–3, 3–5, 5–7 years). For consistency with the primary analysis, we also report the Aalen–Johansen-based restricted mean time lost (AJ-RMTL) ratio by direct integration of the Aalen–Johansen CIF curves.

To assess whether people vaccinated against shingles in April–September 2018 had similar preventive health use before vaccination as those vaccinated in April–September 2017, we compared preventive health indicators in the year before vaccination. We restricted the primary cohorts to individuals with a healthcare encounter 1–2 years before vaccination, that is, between April 2015 and March 2016 for those vaccinated in April–September 2017, and between April 2016 and March 2017 for those vaccinated in April–September 2018. We then rematched the cohorts on the same covariates as above, but measured only before this earlier healthcare encounter, to avoid matching on healthcare use during the year immediately preceding vaccination.

We examined several indicators of preventive health use, including influenza and pneumococcal vaccination, prescriptions for statins, antihypertensives, and antidiabetics, and breast and bowel cancer screening. However, some of these may vary across calendar periods for reasons unrelated to shingles vaccination timing, limiting their value for this comparison. To identify indicators unaffected by such secular trends, we applied the same approach as above, comparing their frequency in the year before TDaP vaccination received in April-September 2018 versus April-September 2017. Only influenza and pneumococcal vaccination showed no systematic difference between the two periods, so we used these as indicators, together with a general code for preventive medicine services (CPT codes 1013829 and 1013859). Using the same approach, we also compared the risk of the primary cardiovascular endpoint in the year before shingles vaccination between the 2017 and 2018 cohorts to assess whether they were on similar cardiovascular health trajectories before vaccination.

Similarly, we assessed preventive healthcare use between cohorts during the follow-up. We considered the same preventive healthcare indicators as above and selected those that showed no secular trends (as detected by comparing cohorts who received TDaP vaccines). This led to the selection of bowel cancer screening (ICD-10 CM Z12.11) and antihypertensive prescription (VA CV100 or CV200 or CV800 or CV701 or CV805), together with a general code for preventive medicine services (CPT codes 1013829 and 1013859).

In the Results:

The association remained similar when accounting for death as a competing risk (and there was no association with non-cardiovascular death; Table 1 and Supplementary Table 3); when the follow-up period was entirely before the COVID-19 pandemic (Supplementary Table 4); when the amount of follow-up time was matched between cohorts (Supplementary Table 5); and when different time windows were used to ascertain covariates (Table 1). In the year before vaccination, the two cohorts did not differ in preventive healthcare use and in the risk of cardiovascular events (Supplementary Table 6). Likewise, there was no significant difference between cohorts during follow-up in terms of preventive health indicators (Supplementary Table 7).

And in the Discussion:

However, the absence of association with a negative control outcome, the lack of difference in clinical trajectories in the year before vaccination, the similarities in preventive healthcare use during follow-up, and the similar risk of non-cardiovascular death provide support for an absence of overt unmeasured confounders.

Modification of the text

In addition, we now make the assumptions behind a causal interpretation of the findings, and the possibility of residual confounding more explicit at multiple places in the manuscript; including in the Introduction:

Because there is no obvious reason for systematic differences between people who were vaccinated on either side of this time cut-off, this design mitigates biases that affect conventional cohort studies.

In the Methods:

Adjustment for these covariates seeks to achieve conditional exchangeability of the cohorts. Importantly, it cannot achieve exchangeability if there are unmeasured confounders (Supplementary Note 3).

In the Discussion:

Third, while our study is a natural experiment (in that treatment allocation is probabilistic and dependent on an external factor[15]) and thus less prone to bias than conventional cohort studies, it does not imply that associations are causal. Causality requires additional assumptions (outlined in Supplementary Note 3 alongside supporting evidence and remaining uncertainties). In particular, while population drifts in measured characteristics (e.g. age) were adjusted for, changes in unmeasured characteristics cannot be excluded. Such unmeasured confounders are plausible given that the transition from the live to the recombinant vaccine followed updated recommendations from the Advisory Committee on Immunization Practices which might have influenced who got vaccinated. Early divergence of Kaplan-Meier curves, while biologically plausible, can be a sign of unmeasured confounding (although later convergence of these curves is incompatible with ongoing influence of time-invariant confounders). However, the absence of association with a negative control outcome, the lack of difference in clinical trajectories in the year before vaccination, the similarities in preventive healthcare use during follow-up, and the similar risk of non-cardiovascular death provide support for an absence of overt unmeasured confounders.

We have also added a section in the Supplement (Supplementary Note 3) outlining the five key assumptions behind a causal interpretation of instrumental variable analyses and the evidence we provide for each, as well as remaining uncertainties.

We believe that the study is therefore more robust but also more nuanced than the original version, and we thank the reviewer for their helpful suggestion.

Stats analyses
Appear appropriate.

Suggested improvements

1. The underlying biological hypothesis is not fully clear. On reading the first part of the main text I'm just not clear at what quasi-randomisation is getting at here as an exposure. Both vaccines prevent zoster, so it is not obvious why the recombinant vaccine would reduce CVD risk whereas the live vaccine would not. Please clarify the proposed mechanism and whether the expectation is a different effect between vaccine types or a general effect of zoster vaccine. Outline this more clearly in the introduction as well as revisiting it in the discussion. I would want to avoid the perception that the live vaccine is worthless in countries/areas where recombinant vaccine is not available.

We agree that a note of clarification was needed in the Introduction. As outlined above, the effect we estimated is the difference in cardiovascular events between recipients of the two shingles vaccines. However, since the live vaccine has been shown not to differ from the unvaccinated population in terms of cardiovascular risks, we hypothesise that the mechanisms underpinning the effect must not directly involve prevention of shingles but rather involve immune and endothelial changes brought upon by the recombinant vaccine specifically.

In the Introduction, we now write:

*By comparing cohorts who have both chosen to be vaccinated against shingles (**with the live vaccine serving as a control given its lack of association with cardiovascular outcomes[5]**), this study design also eliminates the healthy-vaccinee bias.*

In the Discussion, we had written:

Although prevention of shingles might partly explain the effect (given the association between shingles and cardiovascular risk[12] and the greater efficacy of the recombinant vaccine), this explanation appears unlikely for three reasons. […] Third, a natural experiment of live attenuated shingles vaccination compared to no vaccination did not show reduced risks of heart disease or stroke[5]. A more plausible explanation is that the recombinant vaccine induces immune and endothelial changes that are cardioprotective.

Of note, the live vaccine is no longer available anywhere in the world as it has now been discontinued globally.

2. Is it possible to provide stronger evidence that cohorts around the transition are similar?

As detailed in response to the reviewer's first question, we have now conducted seven additional analyses providing stronger evidence that cohorts around the transition are similar.

3. Abstract - “Increased diagnosis free time” is technically correct but not the most natural way to think about the main result. Most readers will find “reduced risk” or “lower incidence” easier to interpret, especially in an abstract. Just a suggestion.

We agree this is not the most natural. Equally, risk and incidence have specific meanings and using them here would be inaccurate. We therefore used ‘burden’ instead, and the sentence now reads:

The recombinant vaccine was associated with a 9% decrease in cardiovascular burden over 7 years.

4. I suggest tone down mechanistic and causal language. Notably, I think the dementia paper is more cautious about its limitations. It states plainly that causality cannot be demonstrated, whereas the language in this paper is a bit more causal e.g. “starts to provide causal evidence”.

We fully agree that some language might have lacked nuances. We have now made several key changes to the manuscript, including:

1. In the abstract, we have changed “To address these biases” by “To mitigate these biases” to reflect the fact that residual biases might still be present.

2. In the introduction, we have removed the statement “To start to provide causal evidence for the cardiovascular effect” and “Because there is no obvious reason for systematic differences between people who were vaccinated on either side of this time cut-off, vaccination timing serves as a quasi-random assignment that minimises confounding.” Instead we have now written:

   Because there is no obvious reason for systematic differences between people who were vaccinated on either side of this time cut-off, this design mitigates biases that affect conventional cohort studies.

3. In the Discussion, we have replaced the sentence “By contrast, our natural experiment minimises these biases and starts to provide causal rather than correlational evidence for a cardiovascular protective effect of the recombinant shingles vaccine” by the following:

   By contrast, our natural experiment mitigates these biases and provides more robust evidence for a cardiovascular protective effect of the recombinant shingles vaccine.

4. In the Discussion, we also explicitly highlight the need for additional assumptions to make causal claims, which we detail in a new Supplementary Note 3:

   Third, while our study is a natural experiment (in that treatment allocation is probabilistic and dependent on an external factor[15]) and thus less prone to bias than conventional cohort studies, it does not imply that associations are causal. Causality requires additional assumptions (outlined in Supplementary Note 3 alongside supporting evidence and a discussion of remaining uncertainties). In particular, while population drifts in measured characteristics (e.g. age) were adjusted for, changes in unmeasured characteristics cannot be excluded.

We believe these nuances significantly help contextualise the findings and the strengths and weaknesses of the study design.

5. As in the dementia paper, the authors suggest that these findings could lead to a more definitive RCT. I do not object to this idea, but I found it unclear what the proposed randomisation would actually be, given that randomisation to no vaccine would be ethically difficult.

This is feasible in countries where vaccine roll-out has not started yet or when it has been limited to specific age groups. Two trials are actually now being set up for dementia (and one of them also includes cardiovascular outcomes): ClinicalTrials.gov ID NCT07485283 and NCT07502560.

Reviewer #3

This is a nicely written paper describing a carefully conducted study that used a natural experiment created by the rapid transition from the live attenuated to the recombinant shingles vaccine in the USA, comparing the incidences of a composite cardiovascular endpoint among adults aged ≥60 years vaccinated immediately before versus immediately after this transition. The recombinant vaccine was associated with a 9% increase in cardiovascular diagnosis-free time over 7 years. Several approaches were used to reduce or assess confounding, and the results are rather robust.

We thank the reviewer for pointing out the strengths of our study.

Negative controls are mentioned but not included in the paper, which they should be.

We wonder whether the reviewer has missed the part in the paper where this is shown. The results for the negative control outcome (a null association with an RMTL ratio of 1.07, 95% CI 0.97 – 1.17, P=0.16) were provided in Table 1 (under 'Robustness analyses' – 'Composite negative control outcome'). In the Results we had written:

As expected, individuals who predominantly received the recombinant vaccine were at a lower risk of shingles and a similar risk of negative control outcome than those who predominantly received the live vaccine (Table 1 and Extended Data Fig. 4).

Additionally, while it is likely that this study shows a decrease in risk of cardiovascular endpoints from the recombinant vaccine, it is also possible that the recombinant vaccine is not protective and in fact the live attenuated vaccine caused cardiovascular disease and the recombinant vaccine did not. This limitation of the study design should be acknowledged

We appreciate that this might have been a possibility. However, there is now strong evidence from a separate natural experiment showing that the live attenuated vaccine (compared to no vaccine) is not associated with a difference in cardiovascular outcomes[10]. We have now clarified this in the Introduction:

*By comparing cohorts who have both chosen to be vaccinated against shingles (**with the live vaccine serving as a control given its lack of association with cardiovascular outcomes[5]**), this study design also eliminates the healthy-vaccinee bias.*

References

1. Titiunik, R. Natural Experiments. in *Advances in Experimental Political Science* 103–129 (Cambridge University Press, 2021).
2. Sun, J. *et al.* Examining cancer screening disparities by race/ethnicity and insurance groups: A comparison of 2008 and 2018 National Health Interview Survey (NHIS) data in the United States. *PLoS One* **19**, e0290105 (2024).
3. Lin, Y.-L., Wang, S.-I. & Wei, J. C.-C. Effectiveness of recombinant zoster vaccine in reducing herpes zoster incidence and all-cause mortality among patients with rheumatoid arthritis: a retrospective cohort study of 21,046 individuals from TriNetX U.S. Collaborative Network. *EClinicalMedicine* **85**, 103319 (2025).
4. Nadarajan, V., Perry, R. J., Johnson, J. & Werring, D. J. Transient ischaemic attacks: mimics and chameleons. *Pract. Neurol.* **14**, 23–31 (2014).
5. SPRINT Research Group *et al.* A randomized trial of intensive versus standard blood-pressure control. *N. Engl. J. Med.* **373**, 2103–2116 (2015).
6. Taquet, M., Dercon, Q., Todd, J. A. & Harrison, P. J. The recombinant shingles vaccine is associated with lower risk of dementia. *Nat. Med.* **30**, 2777–2781 (2024).
7. Weil, J. *et al.* Prophylactic aspirin and risk of peptic ulcer bleeding. *BMJ* **310**, 827–830 (1995).
8. Quan, H. *et al.* Coding algorithms for defining comorbidities in ICD-9-CM and ICD-10 administrative data. *Med. Care* **43**, 1130–1139 (2005).
9. Aaby, P., Netea, M. G. & Benn, C. S. Beneficial non-specific effects of live vaccines against COVID-19 and other unrelated infections. *Lancet Infect. Dis.* **23**, e34–e42 (2023).
10. Pomirchy, M. *et al.* Herpes zoster vaccination and dementia occurrence. *JAMA* **333**, 2083–2092 (2025).

Recombinant shingles vaccine and lower risk of cardiovascular events

Response to reviewers

Fabiana Corsi-Zuelli, Fang Li, Rachel Upthegrove, John A. Todd, Betty Raman, Paul J. Harrison, Maxime Taquet

We thank the reviewers for their helpful comments on our paper. We are delighted to learn that you are interested in publishing our manuscript.

We have now fully addressed the remaining reviewers' comments. We have kept the wording "natural experiment" to help distinguish our study design from other conventional cohort studies, while keeping the discussion on the limitations of the study and their impact on causal interpretation of our findings. We have also made sure that no causal language is used in the paper. In addition to the occurrences of causal language noted by Reviewer 1, we have also changed the following sentences:

Although prevention of shingles might partly explain the ***association*** *(given the association between shingles and cardiovascular risk*[12] *and the greater efficacy of the recombinant vaccine)*

Further studies should try and elicit whether the protective ***association*** *against IHD is mediated by protection against AF (which could explain the lack of association with peripheral arterial disease) or vice versa.*

Reviewer #1

I would like to thank the authors for their revisions. In my view, however, there are still several major concerns in the way in which the study is framed/described that in my opinion need to be addressed before publication. I will of course defer to the editorial team on these matters but would just like to take this opportunity to describe my point of view and reasoning for these opinions. Please find these below.

- Signs of selection bias.

I argued earlier that a main concern in this study is that outcomes may be driven by selection bias. Extended Data Figure 5 suggests some signs of this. In this figure, the authors present a comparison between Shingrix and a) influenza or b) TDaP vaccinators during the same time-period (April-September 2018). The authors present this comparison as additional evidence of the protective effect of Shingrix. But by their own stated logic, these comparisons are subject to the healthy-vaccinee bias that motivated their primary design in the first place. Notable, however, in these comparisons is that the "protective effect" roughly doubles in magnitude compared to the primary analysis (which attempts to control for vaccine selection). This is exactly what you would expect if selection bias was driving or inflating the estimates. Rather than reinforcing the analyses, these secondary comparisons may actually illustrate the magnitude of confounding that the primary design needs to overcome. I think the authors need to discuss this figure and what it may reveal about healthy vaccinee bias more thoroughly.

We agree that these comparisons are more prone to bias as they are based on conventional matched cohort studies. This might explain the greater effect size since part of the effect might be due to this bias. We have now clarified this in the relevant paragraph:

The recombinant shingles vaccine was also associated with a lower risk of the primary endpoint, and each of its components, when compared with the influenza and TDaP vaccines (Extended Data Fig. 5 and Supplementary Table 8), although this was based on conventional matched cohort comparisons that are more prone to bias. This might explain their greater effect sizes than those seen in the primary analysis.

It is worth mentioning that the populations being compared are different from the main analysis, since after matching, the baseline characteristics might differ from those of the whole population receiving the recombinant vaccine (this can be seen in the cumulative incidences differing among recipients of the recombinant shingles vaccine between the primary analysis and these additional analyses). Consequently, RMTL ratios cannot be directly compared between analyses.

In addition, the differences in baseline characteristics between cohorts before matching would tend to increase (rather than decrease) the risk of cardiovascular outcomes among those vaccinated in 2018 (compared to those vaccinated in 2017), which would therefore bias the results towards the null rather than away from it. For instance, people vaccinated in 2018 were more likely to be older and to have a history of smoking, which are both risk factors for cardiovascular outcomes.

- E-value

To properly test whether outcomes are driven by selection bias needs a causal research design, which this study does not offer. In its place, I would recommend the study reports the E-value (VanderWeele and Ding 2017), which quantifies how strong an unmeasured confounder would need to be to fully explain an observed association. Reporting the E-value would provide needed transparency for an observational study like this (and is recommended by STROBE). Applied to the primary RMTL of 0.91 (95% CI 0.88–0.95), a rough calculation yields an E-value of approximately 1.43 for the point estimate and 1.29 for the confidence interval bound closest to the null. This is a little concerning (E-values <2 are usually seen as fragile, 2-5 as moderate, and >5 as large). An unmeasured confounder associated with both treatment assignment and the outcome by risk ratios of only about 1.3 each could potentially explain away the confidence interval (and a ratio of 1.5 removes the entire effect). Given the known associations between health-seeking behaviour, socioeconomic status, and both vaccination uptake and cardiovascular risk, an unmeasured confounder of this magnitude is fully plausible (especially when taking into account the large pre-matching differences we see in the sample characteristics). Therefore, reporting the E-value would make this vulnerability transparent and allow readers to judge for themselves whether matching on observables is sufficient to remove this threat (and I mean reporting these in the main text, with a small explanation for an uninitiated reader, not buried somewhere in the annex).

We agree that the E-value is a helpful statistic. As far as we are aware, it has not been derived for the RMTL ratio. However, the risk ratio can be interpreted as an approximation of the RMTL ratio. Indeed, if cumulative incidence curves are proportional to each other, so that:

$$F_1(t) = R \times F_0(t),$$

then the RMTL ratio is equal to R and is the ratio of the cumulative incidence (‘risks’) at every time point. If R is not constant, then the RMTL ratio is a weighted average of the ratio of cumulative incidences over time.

Using the formula of the E-value for risk ratios and applying it to RMTL ratios, we have an E-value of 1.42 for the estimate. This means that an unmeasured confounder that is associated with cardiovascular disease with a risk ratio of 1.42 (after adjusting for all other confounders) would also need to be 1.42 more common among those who were vaccinated in 2017 rather than 2018. To contextualise this value, extremely sedentary lifestyle (defined as spending an extra 7 hours a day in sedentary behaviour such as sitting and watching TV compared to the rest of the population) increases the risk of cardiovascular disease with a risk ratio of 1.41. There would also need to be over 42% more people with such sedentary lifestyle among those vaccinated in 2017 than in those vaccinated in 2018 (after adjusting for all other covariates) to have a chance to make our result not significant.

There are two important considerations when interpreting the E-value:

1. It represents a worst-case scenario in that an unmeasured confounder with sufficiently large associations with both the exposure and the outcome may still not be enough to explain away the findings (e.g. if they are not frequent enough).

2. The E-value only depends on the effect size (not how robust it is) and so stating that a particular E-value is “fragile” implies that no effect below a certain cut-off (1.33 in the case of the reviewer’s threshold) can ever be robustly observed, no matter the study design. Instead, we believe E-values should be interpreted alongside the study design.

We have now added this in the main manuscript. In the Results:

The E-value was 1.42 indicating that an unmeasured confounder would need to be 1.42 times more common in those vaccinated in 2017 than in those vaccinated in 2018 and to be associated with a 1.42-fold increased risk of cardiovascular outcomes after matching for all other covariates to have a chance to explain away our finding.

And in the Methods:

The E-value was calculated to ascertain the sensitivity of the primary outcome to unmeasured confounders. Given the absence of formulation of the E-value for RMTL ratios, we used the E-value formula for relative risks given that ratios of cumulative incidence can be interpreted as approximations of RMTL ratios: if the cumulative incidence functions are proportional to each other between cohorts ($F1(t) = R \times F0(t)$), the RMTL ratio is equal to the proportionality constant (R). The E-value represents how strongly an unmeasured confounder would need to be associated with both the exposure and the outcome, after adjusting for all measured confounders, to have a chance to explain away the association (i.e. in a worst-case scenario where the prevalence of the unmeasured confounder maximises its bias).

- Causal language

Abstract (p. 2)

“To mitigate these biases, we exploited a natural experiment created by the rapid transition from the live attenuated to the recombinant shingles vaccine”. “Exploited a natural experiment” implies a quasi-experimental identification strategy. As argued earlier, the design lacks a mechanism for as-if random assignment. More accurate would be: “We compared matched cohorts vaccinated before and after the transition”.

In the revised manuscript, we had carefully qualified the terminology of natural experiment, and cited a published definition of a “natural experiment” by Prof. Titiunik, a leader in the field. We believe the term is fully applicable to our study design. We feel that describing the study in this way helps readers understand how it differs from the existing literature, while our discussion makes clear the assumptions, limitations, and appropriate interpretation of the findings. We also note that neither of the other reviewers shared this reviewer’s concerns in this regard.

For these reasons, we would prefer to retain the term, provided that the editor is comfortable with this approach. Of course, we would be happy to follow editorial guidance on the matter.

Introduction (p. 3)

"By comparing cohorts who have both chosen to be vaccinated against shingles (with the live vaccine serving as a control given its lack of association with cardiovascular outcomes), this study design also eliminates the healthy-vaccinee bias.". "Eliminates the healthy-vaccinee bias" is a strong claim that is, in my view, incorrect. Both cohorts chose to be vaccinated, but the selection processes differed between eras (as argued above). "Reduces" or "attenuates" would be more defensible than "eliminates".

The definition of the healthy vaccinee bias is a systematic difference that occurs when comparing vaccinated with unvaccinated people.[1] As we compared two vaccinated cohorts, this study design indeed eliminated this particular bias. This does not imply that the study design is free of other biases and this is why in the preceding sentence, we had written "this design mitigates biases that affect conventional cohort studies".

Additionally, the parenthesis that the live vaccine is a valid control because of its "lack of association with cardiovascular outcomes" relies entirely on one external study that uses a completely different sample and setting (Pomirchy et al 2025). This is a key inferential step that deserves more than a parenthetical citation. If Zostavax does have some cardiovascular effect (even a small one), then the interpretation of the entire study changes.

We agree and we have now clarified this in the limitations section of the discussion:

First, we did not assess the impact of multiple vaccine doses, as this was incompatible with a natural experiment design. ***For the same reason, we did not compare the recombinant vaccine with no vaccine, instead assuming the live vaccine had no cardiovascular effect (based on another natural experiment[13]). If the live vaccine provides some cardiovascular protection, then our results would be underestimates of the true protective association of the recombinant vaccine.***

"Using a similar natural experiment, we have previously provided evidence that recombinant shingles vaccination may directly lower the risk of dementia."

The identical research design is used in Taquet et al. 2024. It is not a natural experiment there either. Also, note the contradicting tendencies in this sentence: on the one hand "natural experiment" indicates that their design can identify a causal effect, but then they qualify this with "may […] lower the risk", implying that the evidence perhaps is better understood as correlational than causal. I'd suggest dropping "natural experiment" here and just saying that "Using a similar design, we have previously…".

We have changed the text accordingly:

Using a similar design, we have previously provided evidence that recombinant shingles vaccination may lower the risk of dementia[6].

Discussion (pp5-7)

"The reduction of risk is clinically meaningful: if confirmed in clinical trials, a 0.8% absolute difference in cumulative incidences of IHD and HF among adults over 60 years-old would translate into hundreds of thousands of cases prevented in the USA alone.". "Cases prevented" is explicitly causal language and is not motivated with the current research design. The entire sentence also needs to be further motivated. The study only estimates a Shingrix-vs-Zostavax difference. Zostavax is already being phased out globally. The relevant public health question going forward is therefore whether Shingrix protects against cardiovascular diseases compared to no shingles vaccination, which this study does not directly answer. The authors only indirectly assume that Zostavax == no shingles vaccine based on one external study conducted in a different setting. These underlying assumptions and the evidence that supports them should be spelled out more clearly when arguing for the practical public health relevance of the findings.

We only refer to "case prevented" under the hypothesis that these results are "confirmed in clinical trials", which makes causal language appropriate in light of the use of the modal auxiliary "would".

As noted above, we have now amended the discussion to clearly outline our assumption that the live vaccine has no effect on cardiovascular outcomes and the impact of any protective effect on our findings:

First, we did not assess the impact of multiple vaccine doses, as this was incompatible with a natural experiment design. ***For the same reason, we did not compare the recombinant vaccine with no vaccine, instead assuming the live vaccine had no cardiovascular effect (based on another natural experiment[13]). If the live vaccine provides some protection, then our results would be underestimates of the true protective effect of the recombinant vaccine.***

"our natural experiment mitigates these biases and provides more robust evidence for a cardiovascular protective effect of the recombinant shingles vaccine.". "Natural experiment" and "protective effect" both imply causality. Drop these. More motivated for the design is "Protective association".

We have removed this sentence altogether as it largely repeated what was in the introduction and we needed to make space for the additions that the reviewer has requested.

"The stronger association for IHD and HF than for ischaemic stroke may indicate a cardiac-specific effect". "Effect" implies causation. "Association" or "pattern" would be more appropriate.

We have now changed it to "pattern".

"the recombinant vaccine induces immune-endothelial changes that are cardioprotective". "Induces" and "are cardioprotective" both strongly imply causality. This sentence presents a specific biological mechanism as the explanation for an association that had not been shown to be causal. "The recombinant vaccine may induce immune-endothelial changes that could be cardioprotective" would at least signal uncertainty.

We have changed it accordingly:

A more plausible explanation is that the recombinant vaccine may induce immune and endothelial changes that could be cardioprotective.

"Such immune changes might be time-limited, explaining the attenuation of the effect in the second half of the follow-up, and supporting studies investigating the effects of repeated vaccination at regular intervals.". "Explaining the attenuation of the effect" treats the attenuation as a feature of a causal mechanism rather than as a pattern in an observational association. "Consistent with the attenuation of the association" would be more accurate.

We have changed it accordingly:

Such immune changes might be time-limited, consistent with the attenuation of the association in the second half of the follow-up

"our study is a natural experiment (in that treatment allocation is probabilistic and dependent on an external factor) and thus less prone to bias than conventional cohort studies, it does not imply that associations are causal". If the term "natural experiment" is dropped, then this sentence appropriately highlights the limitations of the design. The problem is that it comes after extensive causal language in the preceding paragraphs, which undermines its effectiveness.

As stated above, we would prefer to retain the term "natural experiment" as it applies to our study design and helps distinguish it from other studies while the manuscript clearly discusses the assumptions required to make causal inference. Of course, we would be happy to follow editorial guidance on the matter.

Reviewer #2

The authors have substantially addressed my prior concerns. The revised manuscript is improved, provides additional analyses addressing cohort comparability and residual confounding. It appropriately tones down causal claims.

We are pleased to read that the reviewer found that the additional robustness analyses help strengthen the study and that the tone of the manuscript is appropriate.

One very minor suggestion - "eliminates the healthy-vaccinee bias" could be "substantially mitigates healthy-vaccinee bias" or similar

As mentioned in response to Reviewer 1, the definition of the healthy vaccinee bias is a systematic

difference that occurs when comparing vaccinated with unvaccinated people.[1] As we compared two vaccinated cohorts, this study design indeed eliminated this particular bias. This does not imply that the study design is free of other biases and this is why in the preceding sentence, we had written “this design mitigates biases that affect conventional cohort studies”.

Remarks on code availability:
Its a minimal piece of code that shows the approach for some of the main analyses. Minimal data, which is understandable given that patient level data cannot be disclosed. The code runs as it stands.

We agree that this was limited. We have now complemented it with a script that shows all the primary statistical analyses we conducted, using a synthetic (simulated) dataset so that readers can directly see how we conducted analyses.

Reviewer #3

The authors have responded to my earlier review. This is a valuable study and ready for publication.

We are pleased to read that Reviewer #3 recommends publication of our manuscript.

References

1. Nelson, J. C., Jackson, M. L., Weiss, N. S. & Jackson, L. A. New strategies are needed to improve the accuracy of influenza vaccine effectiveness estimates among seniors. *J. Clin. Epidemiol.* **62**, 687–694 (2009).
